# Optimal Combinations of Broadly Neutralizing Antibodies for Prevention and Treatment of HIV-1 Clade C Infection

**DOI:** 10.1371/journal.ppat.1005520

**Published:** 2016-03-30

**Authors:** Kshitij Wagh, Tanmoy Bhattacharya, Carolyn Williamson, Alex Robles, Madeleine Bayne, Jetta Garrity, Michael Rist, Cecilia Rademeyer, Hyejin Yoon, Alan Lapedes, Hongmei Gao, Kelli Greene, Mark K. Louder, Rui Kong, Salim Abdool Karim, Dennis R. Burton, Dan H. Barouch, Michel C. Nussenzweig, John R. Mascola, Lynn Morris, David C. Montefiori, Bette Korber, Michael S. Seaman

**Affiliations:** 1 Theoretical Division, Los Alamos National Laboratory, Los Alamos, New Mexico, United States of America; 2 Santa Fe Institute, Santa Fe, New Mexico, United States of America; 3 Division of Medical Virology, Institute of Infectious Diseases and Molecular Medicine, University of Cape Town and NHLS, Cape Town, South Africa; 4 Center for Virology and Vaccine Research, Beth Israel Deaconess Medical Center, Boston, Massachusetts, United States of America; 5 Department of Surgery, Duke University Medical Center, Durham, North Carolina, United States of America; 6 Vaccine Research Center, NIAID, NIH, Bethesda, Maryland, United States of America; 7 University of KwaZulu-Natal, Durban Department of Immunology and Microbial Science, Durban, South Africa; 8 Centre for the AIDS Programme of Research in South Africa (CAPRISA), University of KwaZulu-Natal, Durban, South Africa; 9 The Scripps Research Institute, La Jolla, California, United States of America; 10 Laboratory of Molecular Immunology, The Rockefeller University, New York, New York, United States of America; 11 National Institute for Communicable Diseases (NICD), NHLS, University of the Witwatersrand, Johannesburg, South Africa; Miller School of Medicine, UNITED STATES

## Abstract

The identification of a new generation of potent broadly neutralizing HIV-1 antibodies (bnAbs) has generated substantial interest in their potential use for the prevention and/or treatment of HIV-1 infection. While combinations of bnAbs targeting distinct epitopes on the viral envelope (Env) will likely be required to overcome the extraordinary diversity of HIV-1, a key outstanding question is which bnAbs, and how many, will be needed to achieve optimal clinical benefit. We assessed the neutralizing activity of 15 bnAbs targeting four distinct epitopes of Env, including the CD4-binding site (CD4bs), the V1/V2-glycan region, the V3-glycan region, and the gp41 membrane proximal external region (MPER), against a panel of 200 acute/early clade C HIV-1 Env pseudoviruses. A mathematical model was developed that predicted neutralization by a subset of experimentally evaluated bnAb combinations with high accuracy. Using this model, we performed a comprehensive and systematic comparison of the predicted neutralizing activity of over 1,600 possible double, triple, and quadruple bnAb combinations. The most promising bnAb combinations were identified based not only on breadth and potency of neutralization, but also other relevant measures, such as the extent of complete neutralization and instantaneous inhibitory potential (IIP). By this set of criteria, triple and quadruple combinations of bnAbs were identified that were significantly more effective than the best double combinations, and further improved the probability of having multiple bnAbs simultaneously active against a given virus, a requirement that may be critical for countering escape *in vivo*. These results provide a rationale for advancing bnAb combinations with the best *in vitro* predictors of success into clinical trials for both the prevention and treatment of HIV-1 infection.

## Author Summary

In recent years, a new generation of monoclonal antibodies has been isolated from HIV-1 infected individuals that exhibit broad and potent neutralizing activity when tested against diverse strains of virus. There is a high level of interest in the field in determining if these antibodies can be used to prevent or treat HIV-1 infection. Because HIV-1 is adept at escaping from immune recognition, it is generally thought that combinations of multiple antibodies targeting different sites will be required for efficacy, much the same as seen for conventional antiretroviral drugs. How many and which antibodies to include in such combinations is not known. In this study, a new mathematical model was developed and used to accurately predict various measures of neutralizing activity for all possible combinations having a total of 2, 3, or 4 of the most promising antibodies. Through a systematic and comprehensive comparison, we identified optimal combinations of antibodies that best complement one another for enhanced anti-viral activity, and therefore may be most effective for the prevention or treatment of HIV-1 infection. These results provide important parameters that inform the selection of antibodies to develop for clinical use.

## Introduction

The ability to elicit potent broadly neutralizing antibodies through immunization remains an elusive goal in the development of an effective HIV-1 vaccine [1]. This has motivated major efforts over the past 6 years to isolate and characterize Env-specific antibodies from HIV-1-infected individuals who exhibit broad and potent serum neutralizing activity [2–4]. Through technological advances in single cell sorting of antigen-specific memory B cells [5–11], high-throughput antibody cloning and screening methods, numerous novel monoclonal antibodies have since been isolated, some of which exhibit exceptional neutralization breadth and potency when tested *in vitro* against large panels of diverse HIV-1 isolates [7, 9–20]. Identification of the epitope targets of these bnAbs has dramatically expanded our knowledge regarding sites of common vulnerability on the Env spike [21]. Major epitope targets include the CD4bs [5, 11, 16, 19, 22–27], a glycan-dependent site in variable region 3 (V3) of gp120 [9, 17, 28–31], a V1/V2 glycan-dependent quaternary site on the apex of the Env trimer [9, 10, 12, 32–37], the MPER [15, 38–41], and epitopes bridging both gp120 and gp41 [13, 14, 18, 42]. The hope remains that characterization of these epitope targets and efforts to elucidate the pathways of bnAb development *in vivo* will eventually result in the rational design of novel immunogens and immunization strategies for eliciting such antibodies through vaccination [12, 16, 24, 43–46]. However, a more immediate potential exists for using bnAbs in clinical settings of passive transfer for the prevention and/or treatment of HIV-1 infection.

In support of preventative modalities, pre-clinical studies in non-human primates (NHP) have demonstrated that passive transfer of bnAbs can confer sterilizing protection against high dose mucosal challenges with chimeric simian-human immunodeficiency viruses (SHIVs) [23, 47–53]. Studies in NHP and humanized mice have further investigated the therapeutic potential of bnAb infusion in the setting of established viral infection, and demonstrated that transfer of single bnAbs can result in a transient decline in plasma viremia, reduction of proviral DNA, and in some cases extended control of viral replication [53–56]. However, viral rebound generally occurs once the concentration of transferred antibody decays below the therapeutic range, and the emergence of neutralization resistant escape variants is often observed. Similar observations were recently described in a phase I clinical study evaluating passive infusion of the CD4bs bnAb 3BNC117 in HIV-1 infected individuals [57]. While escape from antibody monotherapy remains a concern, additional data from animal model studies have shown that therapeutic strategies employing combinations of bnAbs to simultaneously target different epitopes on the Env spike can impede viral rebound and escape, and exert sustained control of viral replication [53–55]. Thus, for bnAbs to be effectively employed for treatment of HIV-1 infection, combinations of multiple antibodies will likely be required to confront the extraordinary diversity of the virus and its ability to escape from selective immune pressure.

Recent studies of *in vitro* neutralization have established that combinations of bnAbs targeting distinct epitopes can act in a complementary and additive manner, and exhibit improved neutralization breadth and potency compared to single bnAbs [58–60]. In the study by Kong et al., it was shown that the breadth and potency of bnAb combinations could be reliably predicted using an additive model, with consistent patterns of minor non-additive interactions for particular bnAb combinations, either antagonistic or synergistic [60]. Certain double, triple and quadruple bnAb combinations were found to achieve 89 to 100% coverage when tested against a large diverse multiclade virus panel. However, due to the complementary nature of the bnAb combinations, in many cases increased breadth was due to only a single bnAb in the mixture exhibiting neutralizing activity against a given virus. In a clinical setting, such a bnAb combination would in essence be the equivalent of single antibody monotherapy against a substantial fraction of viruses, which would have a greater opportunity for escape. Thus, for treatment of HIV-1 infection, it may be advantageous to use bnAb combinations that offer the best potential for active coverage of most viruses by two or more antibodies.

For bnAb immunotherapy in the setting of chronic infection, viral clearance is the most desirable outcome, albeit challenging to achieve. Thus, more complex options are being considered, such as including combinations of the most potent bnAbs together with latency reversing agents (LRAs) and standard antiretroviral drug treatment [61–63]. For such strategies to be beneficial, bnAbs will need to be effective at three levels. First, they will need to neutralize the diversity of viruses circulating in the population targeted for treatment. Second, they will need to effectively neutralize the complex within-host quasispecies that develop during chronic HIV-1 infection. And finally, they should be effective against the full spectrum of expressed forms of Env on any given virion. It has been observed that some bnAbs exhibit neutralization curves that plateau well below 100% when tested against particular Env pseudoviruses *in vitro* [10, 13, 64, 65]. This well-established behavior is surprising given the genetically clonal nature of viruses used in these assays, and could possibly stem from post-translational variation in the glycosylation patterns or alternate variable loop and structural configurations of expressed Env [13, 65–68]. It is a concern that such incomplete neutralization may pose a severe limitation for achieving the desired therapeutic efficacy *in vivo*. Thus, an ideal immunotherapy candidate antibody combination should maximize the genetic and antigenic spectrum of viruses that are potently neutralized, while minimizing the impact of incomplete neutralization.

A key question that remains is how many bnAbs will be required for long term beneficial effects in a preventative or therapeutic setting, and which combinations of bnAbs will provide the most potent and active coverage for testing in human clinical trials. Over the past several years, multiple bnAbs for each major epitope have emerged as viable candidates based on extensive *in vitro* and pre-clinical animal model testing. Given the tremendous resources required to move even a single candidate bnAb forward into human clinical trials, rational decisions must be made to select single antibodies, bivalent antibodies, or components of bnAb combinations that will theoretically provide the highest potency and coverage against the diversity of circulating HIV-1. As bnAb clinical efficacy studies are currently being planned for conduct in southern Africa, coverage and potency of bnAbs against the HIV-1 clade C viruses that dominate the epidemic in that region is of considerable interest.

Here we utilized a newly described panel of 200 acute/early clade C HIV-1 Env pseudoviruses to assess the breadth and potency of 15 of the most promising bnAb candidates targeting four major epitopes of HIV-1 Env. A mathematical modeling approach was developed that increased the accuracy in predicting neutralization titers of bnAb combinations. We experimentally validated the improved accuracy of this model, and then used it to predict the behavior of all possible 2, 3, and 4 bnAb combinations using data derived from single bnAb testing. Using these predictions, we compared the performance of a comprehensive spectrum of potential bnAb combinations, and identified those that provide the most optimal potency, breadth, complete neutralization, and active coverage.

## Results

### Potency and breadth of single bnAbs against a 200 clade C Env pseudovirus panel

**Fig 1:**
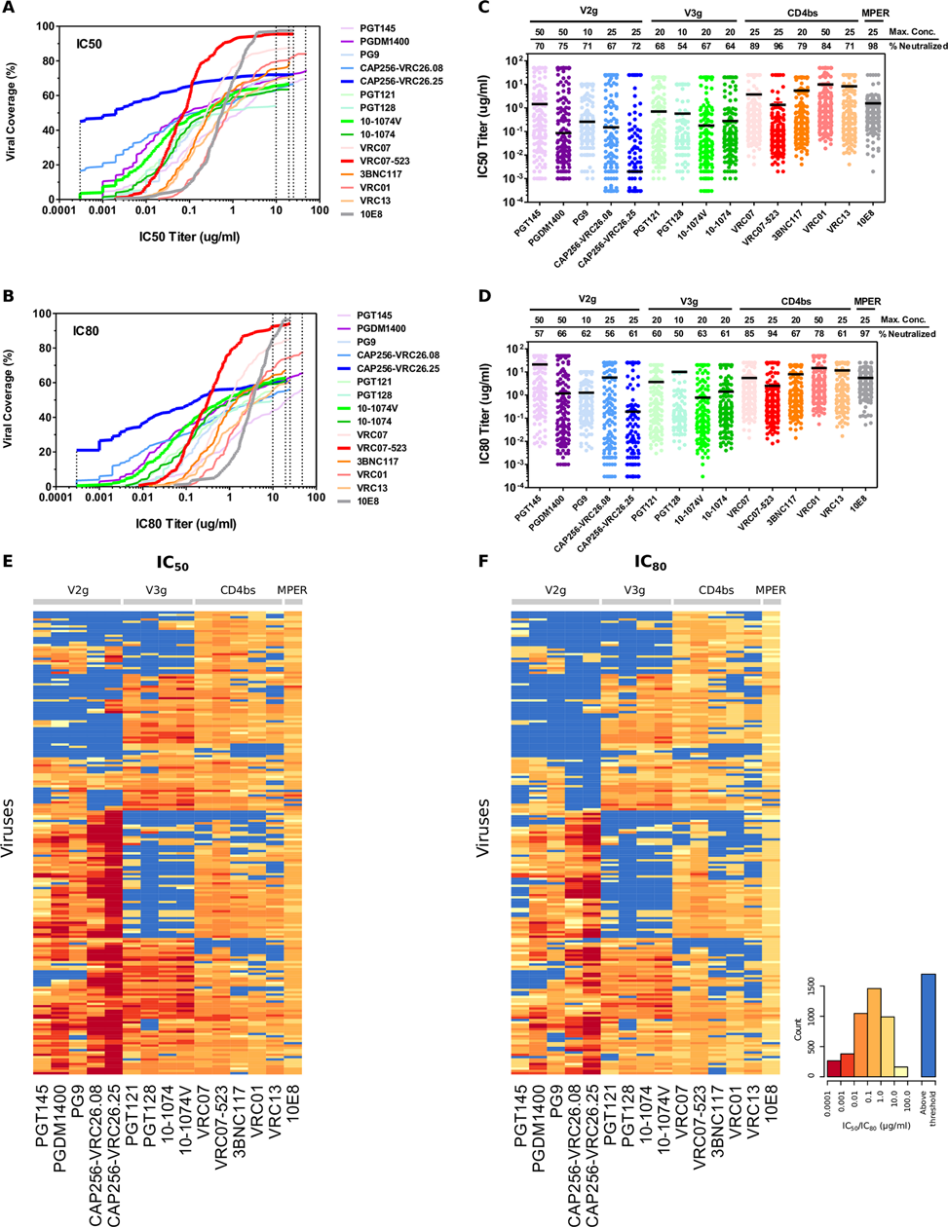
Neutralization activity of bnAbs against clade C virus panel. Potency-breadth curves are presented for both IC_50_ (A) and IC_80_ (B) titers. BnAbs are color coded and grouped by target epitopes. Bold lines indicate bnAbs that were best in class for V2-glycan (V2g), V3-glycan (V3g), CD4bs, and MPER epitopes. Dashed vertical lines indicated the lowest and highest concentration tested. Neutralization data are also presented as scatter plots of IC_50_ (C) and IC_80_ (D) titers in which each virus is represented by an individual dot. The highest concentration tested for each bnAb and the percentage of viruses neutralized are indicated. Solid bars represent median titers. Heat maps of IC_50_ (E) and IC_80_ (F) were generated using the Heatmap tool on the Los Alamos HIV Database. In the heatmaps, rows represent viruses, and columns represent bnAbs. The darker hues indicate more potent neutralization, and blue (for contrast) indicates the virus had IC_50_ or IC_80_ above threshold, unable to reach this level of neutralization at the highest concentration of bnAb tested. The order of viruses is same in panels E and F.

A panel of bnAbs targeting HIV-1 Env was used to assess and compare the breadth and potency of neutralization against acute/early clade C Envs. Fifteen bnAbs were selected that target four distinct epitope regions: the CD4 binding site (CD4bs: 3BNC117, VRC01, VRC07, VRC07-523, VRC13) [11, 19, 23, 69, 70], the V3-glycan supersite (V3g: 10–1074, 10-1074V, PGT121, PGT128) [9, 17], the V1/V2-glycan site (V2g: PG9, PGT145, PGDM1400, CAP256-VRC26.08, CAP256-VRC26.25) [9, 10, 12, 20, 32], and the gp41 MPER epitope (10E8) [15]. BnAbs were tested against a panel of 200 clade C HIV-1 Env pseudoviruses using the validated luciferase-based TZM-bl assay. This virus panel consists of viruses isolated from individuals in the acute/early stages of infection from five southern African countries, including South Africa, Tanzania, Malawi, Zambia, and Botswana. Serial dilutions of individual bnAbs were tested against each virus using a starting concentration that ranged from 10–50 μg/ml, depending on sample availability at the time of testing. Neutralizing activities were evaluated using potency-breadth curves (the percentage of viruses neutralized versus an IC_50_ or IC_80_ cutoff, Fig 1A and 1B), scatter plots (Fig 1C and 1D) and heatmaps (Fig 1E and 1F). The 5 bnAbs targeting the V1/V2-glycan region neutralized between 67–75% of viruses with positive IC_50_ titers, and the 4 bnAbs targeting V3-glycan neutralized 54–68%. When positive, these glycan-dependent bnAbs were strikingly potent. Using the more stringent IC_80_ measure, median IC_80_ titers ranged from 0.003–1.274 μg/ml for V1/V2-glycan and 0.073–0.203 μg/ml for V3-glycan bnAbs (Table A in S1 Text). CD4bs bnAbs tended to exhibit greater breadth (71–96% at IC_50_), but were generally less potent than V1/V2-glycan or V3-glycan antibodies (median IC_80_ titers 0.30–1.58 μg/ml). The MPER directed bnAb 10E8 exhibited lower overall potency (median IC_80_ 3.399 μg/ml), yet had exceptional IC_50_ breadth, neutralizing 98% of viruses. Even the most resistant isolates were sensitive to at least 3 bnAbs, which most often targeted the CD4bs or MPER. Overall, clear differences in potency and/or breadth were observed among bnAbs of the same class (defined here as bnAbs that target the same epitope region). Based on IC_50_ and IC_80_ titers, best-in-class bnAbs were CAP256-VRC26.25 (V2-glycan), 10-1074V (V3-glycan), VRC07-523 (CD4bs), and 10E8 (MPER).

As visualized in heat maps (Fig 1E and 1F), and by hierarchical clustering (Fig A in S1 Text), bnAbs targeting the same epitope region exhibit similar patterns of neutralizing activity, with clear patterns of complementarity between epitope classes. For example, distinct clusters of viruses were resistant to V1/V2-glycan antibodies but sensitive to V3-glycan antibodies, whereas other virus clusters exhibit the opposite phenotype. These data illustrate how different combinations of bnAbs targeting distinct epitopes can complement one another for enhanced coverage against clade C viruses.

### Accurate prediction of bnAb combination neutralization using single bnAb neutralization data

Because it is not practical to assay all combinations of bnAbs against a large panel of viruses, a new method to accurately predict combination bnAb neutralization efficacy using the available large-scale single bnAb neutralization data was developed to facilitate rational decisions for selection of the best bnAb combinations for clinical testing.

In a previous study by Kong et al., the additive model worked well in predicting potency of bnAb combinations using experimental data from single bnAbs [60]. They also found that the experimental bnAb combination data deviated slightly from model predictions. Most combinations performed slightly better than predicted, while a few combinations that included a V3-glycan bnAb performed slightly worse than predicted. The additive model derives from an application of equilibrium mass action kinetics to simplified *in vitro* antibody-virus interactions (S1 Text). This theoretical treatment assumes that single bnAb neutralization curves follow Hill curves with Hill exponents equal to one, and that antibodies act independently with little possibility of multiple antibodies inhibiting the same virion. The first assumption of a unit Hill exponent is largely valid for CD4bs and V3-glycan bnAbs, however, bnAbs targeting the V2-glycan and MPER epitopes frequently exhibit Hill exponents of less than 1 [65, 71, 72].

**Fig 2:**
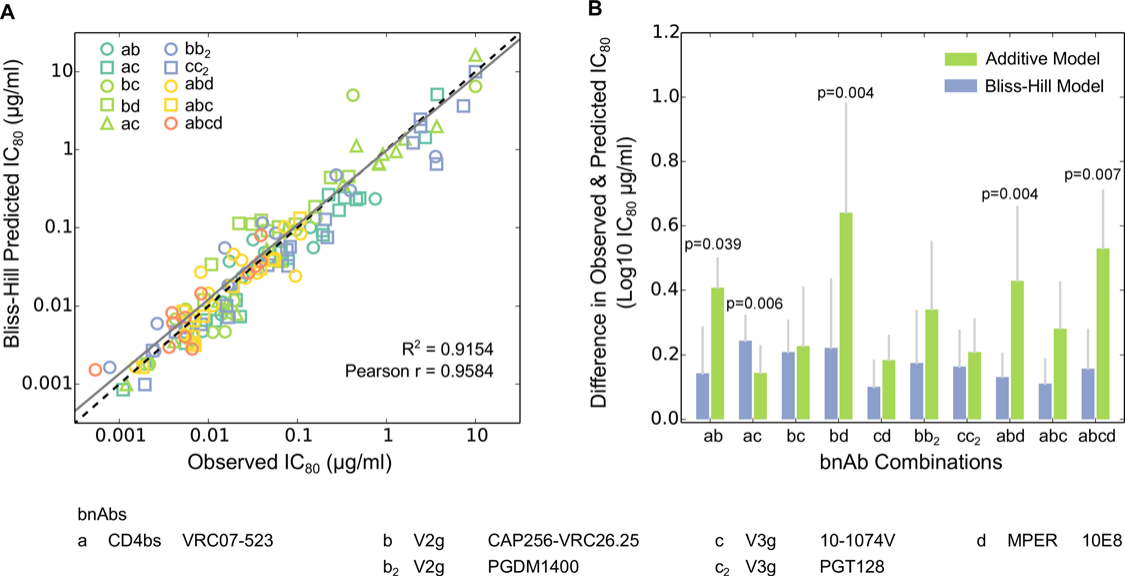
Comparison of Additive and Bliss-Hill models for predicting bnAb combination neutralization scores. Additive and Bliss-Hill models were used to analyze bnAb combination IC_80_ scores for the Clade C Panel. In (A), BH model predictions are plotted against observed IC_80_ values for 20 viruses, with different bnAb combinations (n = 10) shown by different colors and/or symbols. (B) For each bnAb combination tested, the absolute difference between the predicted and the observed Log_10_ IC_80_ values for each virus was calculated using both BH and additive models (Fig D in S1 Text). Median Log_10_ differences using BH model are shown as blue bars and using additive model are shown as green bars, with vertical grey bars representing half the interquartile range. Wilcoxon paired rank test was used to determine whether the Bliss Hill model provides a statistically significantly smaller prediction error for this panel of viruses. Fig D in S1 Text illustrates each of the paired model predictions for the Envs and antibody combinations tested. The additive model often slightly underestimates the observed combination potency, while BH model estimates are closer to the observed. Combinations of bnAbs for which the p-value was smaller than the threshold established by a false discovery rate of q<0.1 are indicated. See Figs C and E in S1 Text for equivalent analysis using the Kong et al. dataset [60].

To overcome these limitations of the additive model, we developed a new model, the “Bliss-Hill model” (BH model). This model combines single bnAb Hill curves (with arbitrary slopes) within the framework of the Bliss independence model for the binding of multiple species of ligands to a substrate [72, 73], and incorporates a correction for multiple ligands independently attaching to the substrate (S1 Text). We tested the BH model by using experimental data from combination bnAb neutralization assays. The assays comprised 10 combinations of 2, 3 and 4 bnAbs (including 2-bnAb combinations with both antibodies targeting similar epitopes, Fig B in S1 Text) assayed against a smaller panel of 20 viruses. The 20 viruses were chosen because they are sensitive to almost all bnAbs tested and comprise a maximized range of IC_80_ titers for the bnAb combinations. The BH model proved highly accurate in explaining the clade C panel bnAb combination data (Fig 2A, R^2^ = 0.9154, Pearson r = 0.9584). Moreover, the BH predictions were closer to the observed data than the additive model for 9 of the 10 combinations tested (Fig 2B, p = 0.021 using Binomial Test), with the only exception being the combination VRC07-523 + 10-1074V. Thus the BH model offered a significant, though modest in magnitude, improvement in prediction accuracy over the additive model. We confirmed this by reanalyzing a larger dataset from Kong et al., and again found the BH model predictions to be highly accurate (R^2^ = 0.9655, Pearson r = 0.9862, Fig C in S1 Text). The BH model performed slightly better than the additive model in all cases, and the difference reached high levels of statistical significance for most of the 2, 3, and 4 bnAb combinations tested. This improvement was due to the systematic trend of BH predictions being more potent than the additive model predictions (Figs D and E in S1 Text), and thus closer to the observed titers since additive model predictions were found to be less potent than the observed titers for most combinations [60].

Nonetheless, for some antibody combinations, experimentally measured IC_80_ titers still showed minor deviations from the BH model predictions (Fig 2, Figs C-G in S1 Text). For a few viruses, the combination IC_80_ titers were 3-fold higher than the most potent bnAb in the combination (Fig D in S1 Text), which is counter-intuitive since both the additive and BH models predict greater potency for combinations relative to the component bnAbs. In such cases we find that the very potent neutralization of a virus by an antibody (particularly CAP256-VRC26.25, Fig D in S1 Text) is somewhat inhibited by the presence of additional antibodies, albeit still resulting in potent neutralization by the combination. Models that incorporated additional parameters based on observed deviations could further improve predictions in some cases (S1 Text, Figs F and G in S1 Text), but the magnitude of deviations were small for most viruses. Furthermore, using deviation modeling with BH model (Fig H in S1 Text) or using additive model (Fig I in S1 Text) did not affect the conclusions below, as the best combinations selected were robust using either model.

### Comparison of neutralization potency and breadth for all potential 2, 3, and 4 bnAb combinations

**Fig 3:**
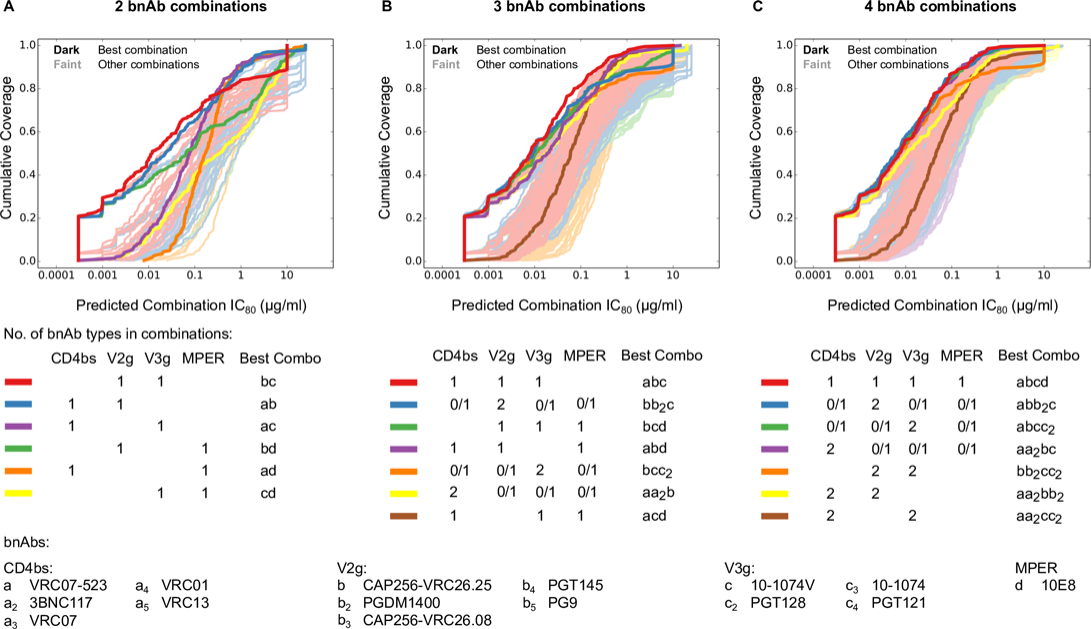
BH predicted IC_80_ potency-breadth curves scores for all candidate 2, 3 and 4 bnAb combinations against the clade C virus panel. Potency-breadth curves for all candidate 2 (A), 3 (B) and 4 (C) bnAb combinations are shown for a total of 1,622 bnAb combinations (81 double-, 431 triple-, 1,110 quadruple-bnAb combinations), using BH model predicted IC_80_ scores. Each combination’s potency-breadth curve is color coded according to the number of bnAbs of different specificities in the combination, e.g. all 4-bnAb combinations that had two V2g bnAbs, and 1 each of other specificities, were assigned to the same category and were color coded blue in (C). The best-in-category bnAb combinations are highlighted in darker colors in A-C, and the others are shown by matched lighter colors. In (B) and (C), “0/1” indicates combinations in which the indicated epitope may or may not have been covered by a representative bnAb. Combinations with a given total number of bnAbs that have 2 bnAbs targeting a single epitope and up to one bnAb targeting other epitopes were grouped together into categories. Such categories are represented as e.g. “2 CD4bs + 0/1 V2g + 0/1V3g + 0/1 MPER” in the figure, which in the case of 4 bnAb combinations, are composed of combination types “2 CD4bs + V2g + V3g”, “2CD4bs + V2g + MPER” and “2CD4bs + V3g + MPER.

Passive and active immunization strategies that aim to protect against the acquisition of HIV-1 infection would benefit from information regarding how many and which bnAb combinations provide optimal coverage and potency. An antibody that may have the best characteristics when considered alone may not have the optimal complementarity when considered for combination bnAb regimens. We predicted the combination scores for all potential 2, 3 and 4 bnAb combinations using the BH model on single bnAb neutralization data for 15 bnAbs against 200 clade C viruses, thus enabling direct comparisons of bnAb combinations. For 2 bnAb combinations, only combinations consisting of bnAbs targeting different epitopes were considered, while for 3 and 4 bnAb combinations, multiple bnAbs targeting the same epitope region were also considered. Predicted potency-breadth curves for all of the 2, 3 and 4 bnAb combinations (1,622 combinations total) are shown in Fig 3.

**Fig 4:**
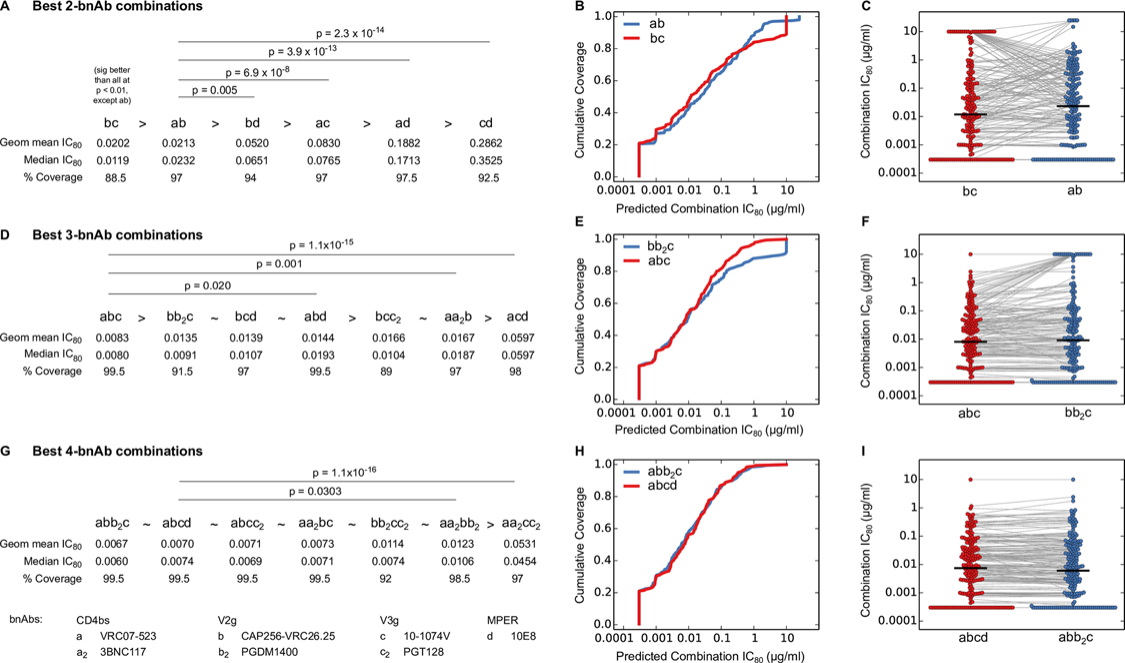
Comparison of best-in-category bnAb combinations for potency and breadth of neutralization. Comparisons of best combinations from each category within the 2- (A-C), 3- (D-F) and 4- (G-I) bnAb combinations are presented. Shown below each combination are its geometric mean and median IC_80_ titer and the percent viral coverage at IC_80_ < 10 μg/ml (A, D, G). Combinations are ordered using geometric mean IC_80_ titers, and a combination is indicated as better than (‘>‘) the proceeding combination when the difference in geometric mean IC_80_ exceeded 0.001 μg/ml, otherwise it is indicated as similar (‘~’). The distributions of IC_80_ scores for the best combinations were compared using a Wilcoxon Rank Sum Test, and only those p-values with q-value < 0.1 are shown. Potency-breadth curves (B, E, H) and distributions of IC_80_ values (C, F, I) for the top 2 combinations are shown. Grey lines in C, F, I connect the predicted IC_80_ values for the same virus.

The combinations were stratified by the number of bnAbs targeting different epitopes (referred to as “categories”, e.g., CD4bs+V2g is a combination of a CD4bs and a V2-glycan bnAb, and V2g(2x)+V3g has two V2-glycan and one V3-glycan bnAbs). Within each category, multiple combinations were possible due to multiple bnAbs targeting the same epitope. Best-in-category bnAb combinations were identified as those with the lowest geometric mean IC_80_ values for the 200 viruses (highlighted in Fig 3 by dark, bold lines). Of note, the area under the IC_80_ potency-breadth curve is negatively, but linearly, and almost perfectly correlated to the Log_10_ geometric mean IC_80_. Thus using either measure gives identical results. The best-in-category combinations were not always clear, as second best combinations were very comparable (e.g. CAP256-VRC26.25 + 10-1074V + PGT128 or PGT121 with geometric mean IC_80_ of 0.007 and 0.0071μg/ml, respectively). Comparisons of best-in-category combinations having the same number of bnAbs are shown in Fig 4.

Best-in-category 2 bnAb combinations had significantly better predicted potency (geometric mean IC_80_ range = 0.02–0.29 μg/ml) and breadth (88.5–97.5% of viruses with IC_80_ < 10 μg/ml), than single bnAbs (geometric mean IC_80_ = 0.17–5.91 μg/ml and breadth = 44–92.5%). The two best-in-category 2 bnAb combinations, CAP256-VRC26.25 (V2-g) with either 10-1074V (V3-g) (geometric mean IC_80_ = 0.020 μg/ml) or VRC07-523 (CD4bs) (geometric mean IC_80_ = 0.021 μg/ml) were significantly better than the other best-in-category 2 bnAb combinations (p < 0.01 and q-value < 0.02) (Fig 4A, 4B and 4C). However, it was unclear which of these two combinations was better, because each pairing had different advantages. While CAP256-VRC26.25 and 10-1074V alone are more potent than VRC07-523 when active (Table A in S1 Text), they have more limited breadth, each neutralizing ~60% viruses at IC_80_ < 10 μg/ml as compared to 92.5% for VRC07-523. Consistent with this, we found that the combination of CAP256-VRC26.25 + 10-1074V missed ~13% of viruses at IC_80_ < 10 μg/ml, while CAP256-VRC26.25 + VRC07-523 missed only ~3%. Thus, while CAP256-VRC26.25 + VRC07-523 was slightly less potent than CAP256-VRC26.25 + 10-1074V, it provides ~10% better coverage.

For 3 bnAb combinations, the best breadth and potency was seen with CAP256-VRC26.25 + 10-1074V + VRC07-523 (Fig 4D, 4E and 4F). This combination, which targets 3 separate epitopes, neutralized 99.5% viruses (all but one in the panel) at IC_80_ < 10 μg/ml, with a geometric mean IC_80_ of 0.0083 μg/ml. The superior performance of this combination draws from the complementary neutralization profiles of the most potent panel bnAbs, CAP256-VRC26.25 and 10-1074V, combined with the broad and potent profile of VRC07-523 (Fig 1). This combination was significantly more potent than most other best-in-category 3bnAb combinations (p < 0.02, q < 0.03). Replacing VRC07-523 with either PGDM1400 or 10E8 in combinations containing CAP256-VRC26.25 + 10-1074V resulted in a small loss of potency and breadth that was not statistically significant. Overall, 3 bnAb combinations showed improved breadth (89 to 99.5% at IC_80_ < 10 μg/ml) and markedly improved potency (geometric mean IC_80_ of 0.008–0.060 μg/ml) than 2 bnAb combinations, with 6 out of 7 best-in-category 3 bnAb combinations predicted to have better geometric mean IC_80_ than the best 2 bnAb combinations.

The two best-in-category 4 bnAb combinations, one targeting 3 epitopes and another targeting 4 epitopes, had comparable potency (geometric mean IC_80_ ~ 0.007 μg/ml) and breadth (99.5% at IC_80_ < 10 μg/ml) (Fig 4G, 4H and 4I), and were more potent and broadly active than 4 bnAb combinations targeting only 2 epitopes (geometric mean IC_80_ of 0.01 to 0.05 μg/ml and breadth 92–98.5% at IC_80_ < 10 μg/ml). Thus bnAb combinations targeting three epitopes showed a significant gain in breadth and potency compared to those targeting two, but the further gain in targeting all four major epitopes, for this panel is negligible. This information is useful to efforts that aim to achieve optimal coverage and potency to protect against the acquisition of infection in passive or active vaccination settings, but does not take into account ease of escape in the setting of passive immunotherapy for active infection.

### Breadth of neutralization by multiple active bnAbs in combination

Combinations of bnAbs are likely to be advantageous in a therapeutic setting not only to maximize potency and breadth but also to minimize the potential for viral escape by targeting multiple epitopes simultaneously [55]. Thus, we investigated the extent of simultaneous neutralization by two or more bnAbs in the best-in-category bnAb combinations at different activity thresholds.

**Fig 5:**
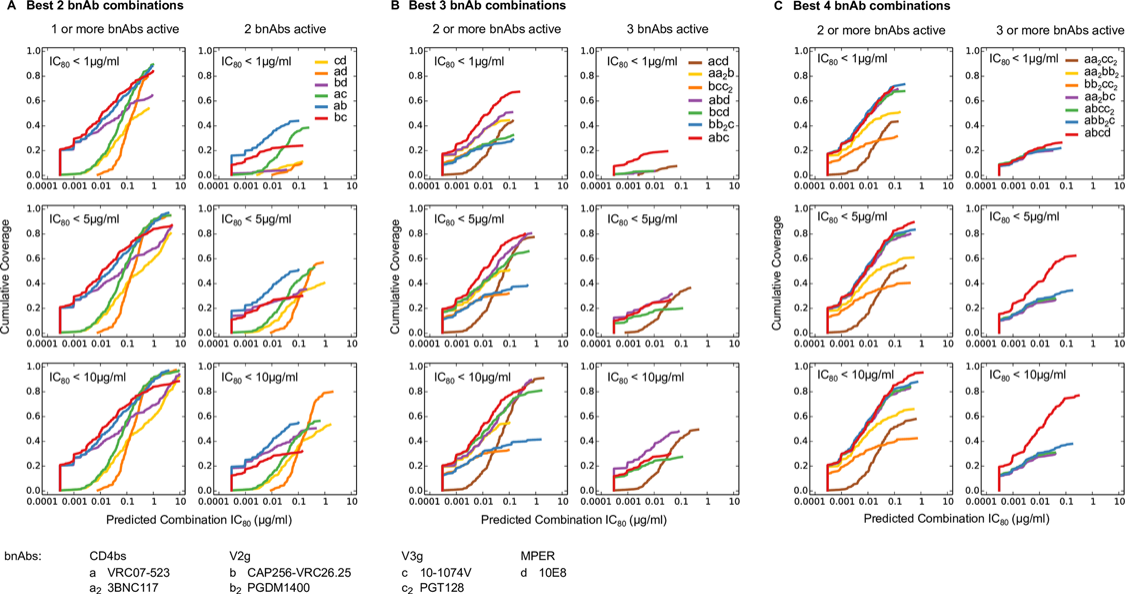
Extent of neutralization by multiple active bnAbs from best-in-category combinations. Modified IC_80_ potency-breadth curves are shown for best-in-category 2, 3, and 4 bnAb combinations. These modified curves measure the fraction of all 200 viruses that are neutralized at predicted combination IC_80_ values, but limited by counting only those viruses that were simultaneously neutralized by at least 1, 2 or 3 bnAbs in the combination. Potency-breadth curves are shown for the best 2 bnAb combinations in which at least 1 or 2 bnAbs were required to be simultaneously active at IC_80_ thresholds of <1 μg/ml, 5 μg/ml or 10 μg/ml (A). Similar potency-breadth curves are shown for the best 3 bnAb (B) or 4 bnAb (C) combinations in which at least 2 or 3 bnAbs were required to be simultaneously active at these IC_80_ thresholds. The modified potency-breadth curves in this figure do not reach the indicated IC_80_ thresholds when 2 or more bnAbs are required to be active because the curves are driven by the IC_80_ value of the more potent active bnAb in the combination, which is often much lower than the activity threshold IC80.

First we quantified the percent of panel viruses actively neutralized by at least 2, 3 or 4 bnAbs in all best-in-category 2, 3 and 4 bnAb combinations at physiologically relevant concentrations. We used IC_80_ thresholds of 1, 5 and 10 μg/ml, which fall in the range of bnAb serum concentrations in HIV-1 infected patients administered a single dose of 1–30 mg/kg of 3BNC117 [57]. For combinations with multiple bnAbs targeting the same epitope class, a modified counting procedure was employed that accounted for overlap in escape-associated mutations (S1 Text). The percent of viruses neutralized by the best bnAb combinations at different thresholds of activity are shown in Table B in S1 Text. We modified the potency-breadth curves for best-in-category bnAb combinations to highlight cases where multiple bnAbs in a combination were simultaneously active (Fig 5). These curves show cumulative coverage of the 200 panel viruses at a given predicted combination IC_80_ value limited by counting only those viruses that were simultaneously sensitive to 2, 3 or 4 bnAbs at single bnAb IC_80_ < 1, 5, or 10 μg/ml.

When the percentage of viruses neutralized by at least 2 bnAbs was considered, the best coverage at our least restrictive threshold within the experimental assay range of IC_50_ <10 μg/ml was 92.5%, 97.5% and 100% for 2, 3 and 4 bnAb combinations, respectively (Table B in S1 Text, Fig 5). This coverage decreased, as expected, to 80%, 91% and 95.5%, respectively, when a more stringent IC_80_ <10 μg/ml threshold was used, and continued to decrease until only 44%, 67.5% and 73.5% coverage was seen, respectively, at our most stringent threshold of IC_80_ <1 μg/ml. The percentage of viruses neutralized when requiring at least three bnAbs in the best-in-category 3 and 4 bnAb combinations to be active was of course even lower at each of these thresholds. Here, the best coverage at the less restrictive threshold of IC_50_ <10 μg/ml was 66.5% and 89% for 3 and 4 bnAb combinations, respectively, and progressively decreased to only 19.5% and 26.5% coverage at the most stringent IC_80_ <1 μg/ml threshold. Poor coverage was seen at all thresholds when all 4 bnAbs in the best-in-category 4 bnAb combinations were required to be active.

Using extrapolated single bnAb neutralization curves (see “BnAb combinations reduce levels of incomplete neutralization” below), we also investigated coverage with multiple active bnAbs using single bnAb IC_80_ < 50 μg/ml and < 100 μg/ml (Fig J in S1 Text). These concentrations roughly approximate the 28 day trough plasma concentrations of passively-administered VRC01 and 3BNC117 in human trials [57, 74] and more closely approximate the range of plasma concentrations that resulted in transient reductions in plasma viremia in patients who received 3BNC117 [57]. We found that the best coverage with 2 bnAbs active at IC_80_ <50–100 μg/ml was 93–100% for 2, 3 and 4 bnAb combinations, and with 3 bnAbs active was 68–92.5% (Fig J and Table B in S1 Text). The overall most potent and broad 2, 3, and 4 bnAb combinations (Fig 4), also had best or close to best coverage with multiple bnAbs active (Fig 5). However, best-in-category combinations that included the exceptionally broad but less potent 10E8 showed superior coverage with multiple bnAbs active at less restrictive thresholds.

### BnAb combinations reduce levels of incomplete neutralization

Neutralization curves for some bnAb/virus pairings can show incomplete neutralization of the genetically clonal virus population [65]. This suggests that a sub-population of virus is resistant to neutralization by the bnAb even at the highest concentrations tested. Given the importance of carbohydrates for many bnAb epitopes, post-translational glycan heterogeneity resulting from incomplete carbohydrate addition or modification may be an important contributing factor to such resistant sub-populations [68]. The inability to neutralize all variants would compromise the use of bnAbs for immunotherapy and may also impede the ability of bnAbs to protect against HIV acquisition. Hence, we investigated the extent of incomplete neutralization of clonal viruses by various bnAb combinations.

**Fig 6:**
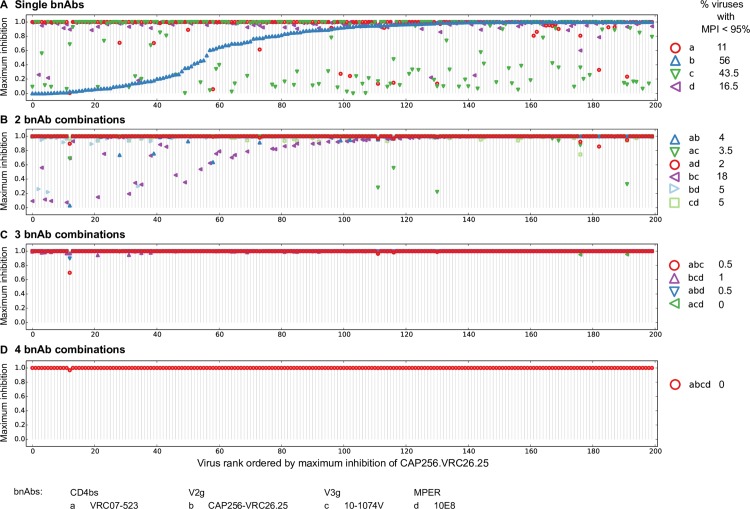
MPI exhibited by single bnAbs and bnAb combinations. The observed MPI for single bnAbs (A) and predicted MPI values for 2, 3 and 4 bnAb combinations (B-D) are shown. Vertical lines indicate each of the 200 panel viruses and are ordered by MPI values for CAP256-VRC26.25 in all panels. For each virus, the MPI for a bnAb or a bnAb combination is represented by a symbol as shown to the right. The percentage of viruses with MPI <95% for each bnAb and bnAb combination is also indicated.

We first analyzed neutralization curves for single bnAbs and bnAb combinations that were experimentally measured in the study by Kong et al. [60]. We could accurately predict the combination maximum percent inhibition (MPI) using the Bliss independence model on single bnAb MPI values (Methods, Fig K in S1 Text, Pearson r = 0.9904, difference between observed and predicted MPI: median = 0.1%, 95% CI = 0–4.5%). Using this model, we then predicted the MPI values for the 2, 3 and 4 bnAb combinations composed of the best single bnAbs against the clade C panel. Experimental MPI values for single bnAbs are shown in Fig 6A (see S1 Text for discussion on different assay starting concentrations for panel bnAbs), and the predicted MPI values for 2, 3 and 4 bnAb combinations are shown in Fig 6B, 6C and 6D, respectively.

Incomplete neutralization was observed against several viruses for all single bnAbs and was frequent for the V2- and V3-glycan bnAbs CAP256-VRC26.25 and 10-1074V, (56% and 44% viruses with MPI < 95%, respectively). A lower frequency of incomplete neutralization was observed with VRC07-523 (11% viruses with MPI < 95%) and 10E8 (16.5%). Encouragingly, the fraction of resistant variants within a single virus preparation was predicted to decrease with increasing number of bnAbs in a combination, indicating that bnAbs tend to be complementary not only in terms of viral sensitivity at the population level, but in terms of the resistant subpopulations of post-translational Env variants. The 2 bnAb combination with the least fraction of viruses incompletely neutralized was VRC07-523 + 10E8 (2%), while VRC07-523 + CAP256-VRC26.25, which had one of the best potency and breadth profiles, had 4% viruses with MPI < 95%. Consistent with the high levels of incomplete neutralization seen with the V2- and V3-glycan bnAbs, a higher extent of incomplete neutralization was predicted for CAP256-VRC26.25 + 10-1074V, where MPI <95% was seen for 18% of viruses. Strikingly, the 3 bnAb combinations had MPI < 95% for only 0.5–1% viruses (n = 1–2 out of 200), and the 4 bnAb combination never had MPI < 95% for any virus. The analysis of experimentally measured MPI from the Kong et al. study also showed similar patterns (Fig L in S1 Text).

Studies of passive bnAbs in humans aim to achieve plasma concentrations that for periods of time exceed 25 μg/ml, a dose commonly tested in our neutralization assays [60]. We therefore experimentally tested the extent of incomplete neutralization at concentrations of up to 100–200 μg/ml against a subset of 24 viruses that were selected based on incomplete neutralization at the lower doses tested (Fig M in S1 Text). Most of these viruses were still incompletely neutralized at the highest concentrations tested (only 1 out of 24 showed 95% or higher neutralization). We then estimated the best-fit Hill curves using data points below 25 μg/ml (Methods, S1 Text) and used these to predict neutralization at the highest concentrations tested for each of these high-concentration assays. The predictions were quite accurate (average root mean square error = 6%, Kendall Tau p = 3.7 x 10^−5^, Fig N in S1 Text). Thus, using this approach, we predicted the MPI at 100 μg/ml for all best-in-class bnAbs (Fig N in S1 Text) and their combinations (Fig O in S1 Text) for all 200 clade C panel viruses. As expected, the fraction of viruses with predicted neutralization less than 95% at 100 μg/ml was reduced compared to the values at 25 μg/ml. Still, we found substantial levels of incomplete neutralization at 100 μg/ml and these results qualitatively recapitulated the above patterns of MPI at 25 μg/ml for single bnAbs and for bnAb combinations.

### Instantaneous inhibitory potential of bnAb combinations

The metric instantaneous inhibitory potential (IIP) measures the log_10_ reduction in a single round of infection events in the presence of a drug. This metric correlates with clinical success of antiretroviral drug combinations, and can be used to characterize their efficacy [75]. Jilek *et al*. found that IIP_ave_ values (average IIP during the dosing interval, given drug pharmacokinetics) of 5–8 logs were necessary for successful antiretroviral therapy. Drug combinations in this range showed a reduction of viral load to <50 RNA copies/ml at 48 weeks in 70% or more of infected individuals. Applying their approach, we calculated the IIP values for the best-in-class single bnAbs and best bnAb combinations for the clade C panel.

**Fig 7:**
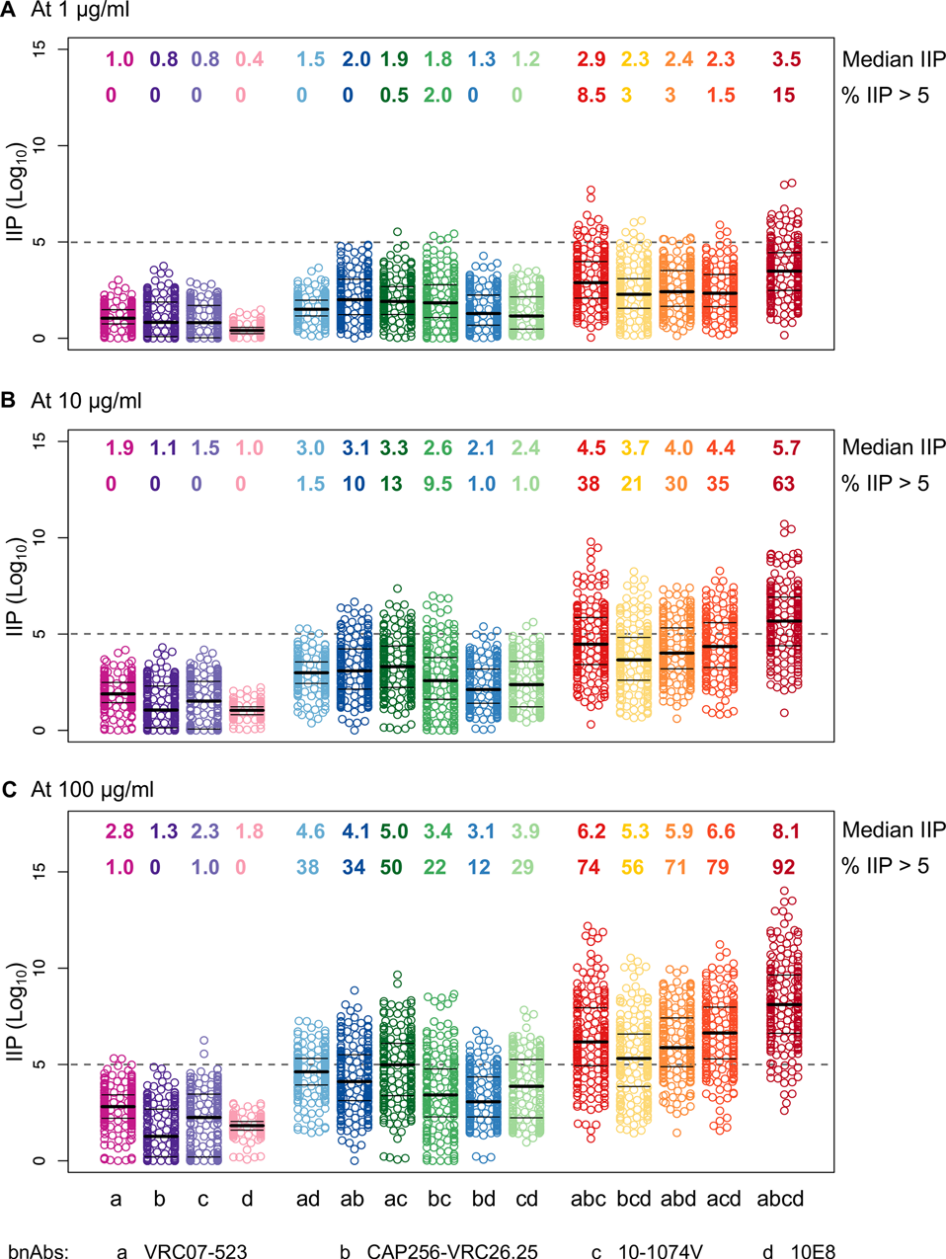
IIP for bnAbs and bnAb combinations. IIP values (log_10_ reduction) are shown for the best-in-class single bnAbs and their combinations at 1 μg/ml (A), 10 μg/ml (B) and 100 μg/ml (C). In each panel, numbers in the top row show median IIP values and in the bottom row show the percentage of viruses with IIP > 5. The dotted horizontal lines are at IIP = 5.

IIP values for single bnAbs were calculated using either the best-fit Hill curves of experimental neutralization data for the best-in-class bnAbs (Fig 7, S1 Text), or estimated Hill curves using IC_50_ and IC_80_ values (Fig P in S1 Text) (with the former expected to yield more accurate predictions since IIP values are critically sensitive to neutralization close to 100%). Using BH model, we calculated the IIP values (Methods) for 2, 3 and 4 bnAb combinations of the best-in-class bnAbs (Fig 7). Since IIP values depend on bnAb concentration, and precise doses and pharmacokinetics of bnAbs are still being established, we analyzed IIP at bnAb concentrations of 1, 10 and 100 μg/ml. The 1 and 10 μg/ml concentrations are within the experimental assay range, whereas results for the 100 μg/ml dose are estimates obtained by extrapolation.

The best-in-class single bnAbs had median IIPs of 0.4–2.8 across viruses, depending on the bnAb and concentration, with CD4bs bnAb VRC07-523 giving the highest value, followed by V3-glycan bnAb 10-1074V (Fig 7, Fig P in S1 Text). The best-in-category bnAb combinations showed higher median IIP values of 1.2–5.0, 2.3–6.6, and 3.5–8.1 for 2, 3 and 4 bnAb combinations, respectively. The 2 bnAb combinations with highest IIP values consisted of VRC07-523 with either CAP256-VRC26.25 or 10-1074V, depending on the concentration. The 3 bnAb combinations with the highest IIP values were VRC07-523 + 10-1074V with either CAP256-VRC26.25 or 10E8, with the latter combination having a slightly better median IIP at 100 μg/ml (median IIP of 6.2 and 6.6, respectively).

Single bnAbs rarely had IIP > 5, the level found to be critical for clinical success of antiretroviral drug combinations [75], while 2, 3 and 4 bnAb combinations had IIP > 5 for 0–50%, 1.5–79%, and 15–92% of viruses, respectively, depending on concentration. The median IIP of the best 3 bnAb combinations exceeded 5 only at 100 μg/ml, while the best 4 bnAb combination had median IIP > 5 at a lower concentration threshold of 10 μg/ml. The range of median IIP values for the best 4 bnAb combination (3.5–8.1) is comparable to the average IIP for some of the currently prescribed antiretroviral triple-drug combinations (IIP ~ 3.5–12) [75].

### Comparison between the best 2, 3, and 4 bnAb combinations

We next systematically compared the best-in-category 2, 3, and 4 bnAb combinations to evaluate the benefit of having combinations with more total antibodies on overall performance using the metrics described above; namely the overall potency-breadth curves (Figs 3 and 4), the number of active bnAbs in the combination (Fig 5), the extent of incomplete neutralization (Fig 6), and IIP values (Fig 7). The relative impact of these metrics on clinical success is unknown and the relevance of each metric might differ for prevention versus treatment of HIV-1 infection, e.g. neutralization by multiple active bnAbs and IIP may be more relevant for latter. Working under the a priori hypothesis that an ideal combination should maximize performance using all four metrics, we chose VRC07-523 + CAP256-VRC26.25, VRC07-523 + CAP256-VRC26.25 + 10-1074V and VRC07-523 + CAP256-VRC26.25 + 10-1074V + 10E8 as the best 2, 3, and 4 bnAb combinations for comparison, respectively. These combinations showed best or near best performance using all four metrics when compared with other combinations with same number of bnAbs.

**Fig 8:**
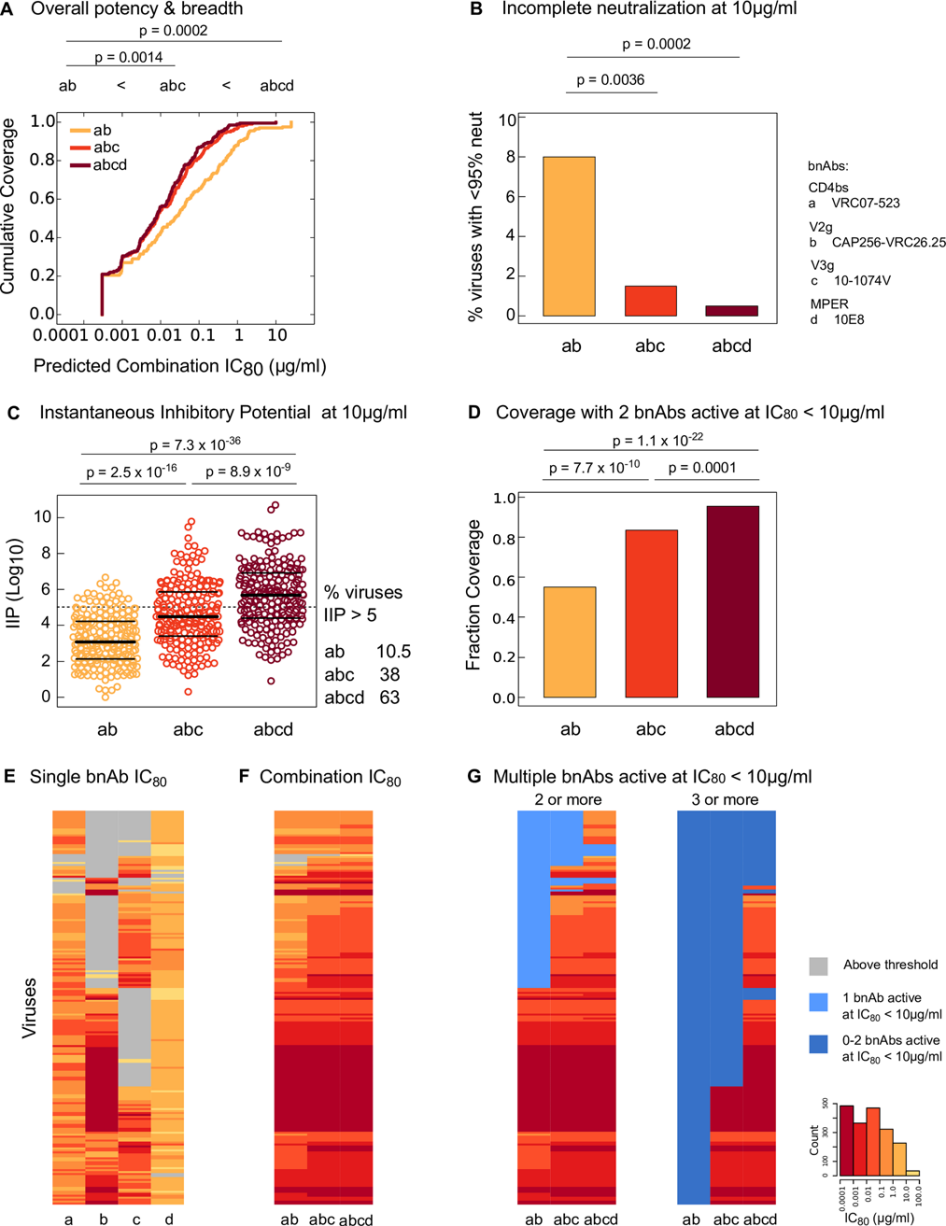
Comparison of best 2, 3 and 4 bnAb combinations. (A) Potency-breadth curves for the best combinations are shown. IC_80_ scores for combinations were compared using Wilcoxon rank sum test. (B) Fraction of viruses (total n = 200) predicted to have < 95% neutralization at 10 μg/ml for the best combinations. Fisher’s exact test was used to calculate the statistical significance. (C) IIP calculated at 10 μg/ml for the best combinations against each of the 200 panel viruses. Statistical significance of the difference in IIP values was found using Wilcoxon rank sum test. (D) Fraction of viruses neutralized by at least 2 bnAbs in the best combinations at single bnAb IC_80_ thresholds of < 10 μg/ml. Fisher’s exact test was used to calculate statistical significance. (E, F, G) show heatmaps of IC_80_ values for the best single bnAbs and the best bnAb combinations respectively. Rows represent viruses, and columns represent single and combination bnAbs. Same ordering of viruses is used in E-G. Darker hues of red indicate more potent neutralization and grey cells indicate IC_80_ above threshold. In (G), blue shades indicate viruses that were neutralized by less than 2 bnAbs (left panel) or by less than 3 bnAbs (right panel) at single bnAb at IC_80_ < 10 μg/ml.

Using overall potency and breadth profiles, the best 3 and 4 bnAb combinations were significantly more potent than the best 2 bnAb combination, with a 2.6–3.1-fold more potent geometric mean IC_80_ (Fig 8A, p < 0.0014), and showed higher breadth of 97–99% versus 87% viruses neutralized at IC_80_ < 10 μg/ml, respectively. The best 3 and 4 bnAb combinations also demonstrated superior performance over the best 2 bnAb combination in limiting the extent of incomplete neutralization (Fig 8B). The fraction of viruses predicted to have < 95% neutralization at 10 μg/ml for 3 (1.5% viruses) and 4 bnAbs (0.5% viruses) was significantly lower than that for 2 bnAbs (8% viruses, p < 0.0036). Similarly, IIP for 3 and 4 bnAb combinations were significantly higher than the 2 bnAb combination (Fig 8C, p < 2.5 x 10^−16^), and showed significantly higher fraction of viruses above the clinically relevant threshold of 5 (p < 1.2 x 10^−10^, Fisher’s exact test). The best 3 and 4 bnAb combinations also showed significant improvement of coverage with at least 2 bnAbs active (Fig 8D and 8G, 28–42% improvement in coverage, p < 7.7 x 10^−10^). The main reason behind the poor coverage of viruses neutralized by 2 bnAbs in the best 2-bnAb combination was the limited breadth of CAP256-VRC26.25, which was included for its potency when positive (Fig 8E–8G).

Four bnAbs were predicted to be similar to 3 bnAbs by some metrics, and significantly better by others. The best 3 and 4 bnAb combinations showed nearly identical distributions of IC_80_ values (Fig 8A), and levels of incomplete neutralization (Fig 8B). In contrast, the best 4 bnAb combination showed significantly higher coverage than the best 3 bnAb combination for both neutralization by at least 2 active bnAbs (improvement in coverage 9.5% using activity threshold of IC_80_ < 10 μg/ml, p = 0.0001), and by at least 3 active bnAbs (improvement in coverage 47%, p = 1.9 x 10^−21^, Fisher’s exact test) (Fig 8D and 8G). Also potentially relevant for success in therapeutic settings, the best 4 bnAb combination showed significantly higher IIP scores (Fig 8C, p = 8.9 x 10^−9^) and significantly higher number of viruses with IIP > 5 than the best 3 bnAb combination (25% more viruses, p = 8.5 x 10^−7^). These results indicate that 4 bnAb combinations may be more effective in preventing viral escape compared to 3 bnAb combinations.

## Discussion

The exceptional breadth and potency of a new generation of bnAbs offers new clinical opportunities for the prevention and/or treatment of HIV-1 infection. Two CD4bs bnAbs, VRC01 and 3BNC117, have already initiated phase I clinical testing in infected subjects, and efficacy studies for the prevention of HIV-1 infection are planned [57, 74]. The most effective approaches will likely employ combinations of bnAbs targeting multiple epitopes on HIV-1 Env to maximize potency and coverage and to impede escape, which may be particularly important in the case of immunotherapy. Prevention of sexual transmission of HIV-1 may represent a relatively easier target for success, as bnAbs at mucosal surfaces at the time of exposure need only to block the infecting virus, while therapeutic approaches need to contend with high levels of replicating virus, complex within-host viral diversity, and established latent viral reservoirs. Given the large number of bnAbs now available against multiple epitope regions of HIV-1 Env, it is of great interest to have experimental measures and predictive models that can be used for evaluating and selecting optimal combinations of bnAbs for clinical development for the prevention and/or treatment of HIV-1 infection.

Among the bnAbs tested here, the best-in-class single bnAbs for potency and breadth against our panel of 200 clade C viruses were CAP256-VRC26.25 (V2-glycan), 10-1074V (V3-glycan) and VRC07-523 (CD4bs) (Fig 1). While 10E8 was the only MPER-directed bnAb tested, it was previously shown to be the most broadly reactive and potent of the known MPER bnAbs against other virus panels [15]. To evaluate various combinations of bnAbs we developed a new model, the Bliss Hill (BH) model, and found it to more accurately predict the breadth and potency of antibody combinations than the additive model (Fig 2, Fig C in S1 Text). We applied the BH model to predict neutralization profiles of over 1,600 possible 2, 3, and 4 bnAb combinations against the 200 clade C viruses using experimental data from the testing of single bnAbs alone (Figs 3 and 4). These predictions allowed us to identify and compare best-in-category bnAb combinations. The overall potency and breadth of neutralizing activity significantly improved as the total number of bnAbs in the combination was increased from 2 to 3, but not from 3 to 4 (Figs 4 and 8). Two best 2 bnAb combinations were identified that demonstrate superior performance in overall potency and breadth. While CAP256-VRC26.25 + 10-1074V was slightly more potent than CAP256-VRC26.25 + VRC07-523, the latter combination exhibited better breadth, and thus may be preferred. The best 3 bnAb combination (CAP256-VRC26.25 + VRC07-523 + 10-1074V) benefitted from combining the complementary potent profiles of CAP256-VRC26.25 and 10-1074V, with the added potency and breadth of VRC07-523. The best 4 bnAb combinations were significantly better than the best 2 but not 3 bnAb combinations. Together, these results demonstrate the substantial benefit bnAb combinations afford when selected to complement and optimize target epitopes, potency, and breadth of coverage. These parameters will be important to consider when selecting bnAb combinations for both prevention and immunotherapy of HIV-1 clade C infection.

We note that 8 of the 15 bnAbs tested here did not show up as a component of best combinations. In most cases these bnAbs exhibited weaker potency and breadth of neutralization than bnAbs in the corresponding epitope class that did show up (Fig 1A). An exception is VRC07, which had a better profile than 3BNC117, yet 3BNC117 and not VRC07 showed up as a component of best combinations. Another exception is PGT121, which was marginally better than PGT128, yet PGT128 and not PGT121 showed up in best combinations. In both of these cases the bnAb in best combinations (3BNC117 and PGT128) had slightly greater potency against sensitive viruses (Table A in S1 Text).

Our analyses further highlight that bnAb combinations, especially those to be used for treating established HIV-1 infection, can be selected to increase the probability of having at least two antibodies in the mixture active against a patient’s virus. While having an increased number of active bnAbs in a combination is desirable, our results illustrate the sobering limitations with even the best bnAbs currently available (Figs 5 and 8). For IC_80_ thresholds of 1–10 μg/ml, the percentage of clade C viruses neutralized was reduced to 44–95.5% when requiring a minimum of 2 bnAbs in the combination to be active. This coverage substantially increased when IC_80_ thresholds of 50 μg/ml or higher were considered (Fig J in S1 Text). Therefore maintaining high *in vivo* antibody concentrations, in plasma and especially in infected tissues, may be key in therapeutic settings, and thus the tissue distribution and *in vivo* pharmacokinetics of individual bnAbs will be critical factors. The coverage of viruses by active antibodies naturally increased with the total number of bnAbs included in a combination, yet even for the best 4 bnAb combination, only 73.5%, 26.5%, and 2.5% of viruses would have either 2, 3, or all 4 antibodies active at a threshold IC_80_ titer of < 1.0 μg/ml, respectively. From these analyses, it becomes apparent that inclusion of a bnAb with better overall breadth (such as 10E8, Figs 1 and 5, Fig J in S1 Text) in a combination may be more advantageous than choosing the most complementary bnAbs with the highest potency. By further analogy to antiretroviral therapy, it is possible that at least 3 agents simultaneously active against the virus will be critical to avoid escape. For the prevention of HIV-1 infection, it may not be quite as critical to have multiple antibodies simultaneously active, as bnAbs at mucosal surfaces need only to block the transmitting virus at the time of exposure. Nonetheless, combinations of at least 2 or 3 bnAbs may provide an advantage for breadth and potency in preventing infection, and should enhance coverage against viral quasispecies from a chronically infected donor.

We also considered the impact of bnAb combinations on limiting the extent of incomplete neutralization of HIV-1 Env pseudoviruses. Combinations with a higher number of bnAbs, in addition to improving breadth and potency across different viruses, also improved the capacity to completely neutralize the expressed forms of an Env within a genetically clonal virus population (Fig 6, Figs L and O in S1 Text). The experimental data suggests that the resistant sub-populations of virions for different bnAbs do not overlap substantially. This complementarity reduces the extent of incomplete neutralization shown by combinations with higher number of bnAbs, an important consideration when selecting optimal bnAb combinations for both prevention and treatment of HIV-1 infection. It should be noted that the pseudoviruses utilized in our study were produced in 293T cell lines, and thus may differ in glycan heterogeneity and susceptibility to incomplete neutralization compared to viruses derived from primary PBMC. However, a recent study comparing clonal viruses grown in either 293T or human PBMC found overall similar trends in levels of incomplete neutralization for individual bnAbs [65]. These data suggest that the complementarity of bnAbs to limit incomplete neutralization will likely prove to be effective for primary PBMC grown viruses as well.

The slopes of *in vitro* neutralization curves for individual bnAbs have been shown to exhibit inherent variability, with bnAbs exhibiting slopes >1.0 predicted to have greater *in vivo* efficacy than classes of bnAbs having slopes ≤1.0 [71]. The metric instantaneous inhibitory potential (IIP), which measures the Log_10_ reduction in infectious events in the presence of drugs/antibodies, is positively correlated with neutralization curve slopes, in that bnAbs with higher slopes are predicted to have IIP values that increase faster with concentration [76]. Here we calculated IIP values for best-in-category bnAb combinations as an opportunity to quantitatively compare their efficacy based on what is seen with antiretroviral drug combinations [75]. Such a comparison between bnAbs and standard antiretroviral drugs comes with several caveats. First, Env is much more variable than the targets of most antiretroviral drugs, making it essential to measure bnAb activity against a large panel of virus variants, whereas IIP values in the Jilek et al. study were calculated for a single virus. Second, because bnAbs can engage in Fc receptor-mediated effector functions [77, 78], the overall *in vivo* efficacy of bnAb combinations might be greater than the neutralization measured *in vitro*. Third, since IIP values depend on the concentration of drug, tissue-wide heterogeneity and pharmacokinetic profiles of bnAbs will be needed for accurate prediction. With these caveats in mind, we found that IIP values for the best 3 and 4 bnAb combinations compare favorably with those of several available antiretroviral drug combinations, for which an IIP threshold of 5–8 was found to correlate with clinical success [75]. While single bnAbs and 2 bnAb combinations had IIP < 5 for most viruses, we found that the best 3 and 4 bnAb combinations had median IIP values > 5 at concentration thresholds of 100 μg/ml and 10 μg/ml, respectively (Fig 7). Thus, using the Jilek et al. criterion, the 3 and 4 bnAb combinations could lead to favorable clinical outcomes, while single and 2 bnAb combinations are less likely to succeed.

It must be emphasized that the results from our analyses do not imply that other bnAb candidates should not be further considered for inclusion in combinations for clinical testing. In fact V3-glycan bnAbs 10–1074 and PGT121 have either started or will soon initiate phase I clinical testing, respectively. Our results do, however, suggest favorable bnAb combinations for future studies, and provide a reasoned way to narrow the otherwise vast array of possible bnAb combinations. We provide modeling strategies that enable quantitative assessment of the neutralization patterns of combinations of bnAbs using several metrics, to better inform selection for clinical use. Yet these *in vitro* measures and modeling results are just a few of the parameters that must be considered when selecting optimal bnAb candidates. The stability, manufacturability, and *in vivo* pharmacokinetics, tissue distribution, and safety profiles are just a few additional key parameters that must also be evaluated when moving bnAb candidates forward in the clinical pipeline.

Our study focused on HIV-1 clade C viruses as the predominant subtype in sub-Saharan Africa where bnAb clinical efficacy studies will likely be conducted, and is a dominant subtype globally. Some bnAb combinations may be more effective against other genetic subtypes, as bnAbs can exhibit variable levels of neutralization breadth among different clades of virus (e.g. many V3-glycan antibodies exhibit more limited breadth against CRF01_AE viruses, and CAP256-VRC26.25 has limited breadth against clade B viruses) [17, 32]. Extensive data sets are available from the testing of individual bnAbs against large standardized panels of viruses from multiple subtypes, and the BH-model presented here may be utilized to thoroughly investigate the question of how viral clade impacts optimal bnAb combinations. We are developing a web-tool, CombiNaber, which will available on the Los Alamos HIV Immunology Database (http://www.hiv.lanl.gov/content/sequence/COMBINABER/combinaber.html). This tool will predict bnAb combination neutralization results from single bnAb neutralization data using either BH or additive models and perform systematic analysis to provide the user with the best candidate combinations for their panel (S1 Text).

In summary, we have assessed optimal bnAb combinations predicted to have greatest success in the prevention and treatment of infection by HIV-1 clade C, taking into account multiple metrics. In addition to evaluating overall potency and breadth, we have also taken into account the number of active bnAbs within a given combination, the impact of combinations in limiting the extent of incomplete neutralization, and to calculate the IIP of bnAb combinations. These latter metrics may be of critical importance when considering the use of bnAbs for the treatment of HIV-1 infection, as they directly relate to confronting the ability of virus to escape from selective immune pressure. Our results indicate that for both the prevention and treatment of HIV-1 infection, combinations with higher numbers of bnAbs are advantageous in providing increased potency, breadth, complete neutralization, and active coverage. Given the tremendous resources required to take each single bnAb forward into clinical testing, our results outline important parameters that can inform the selection of bnAbs with the best indicators of success for clinical development, and stresses the importance of considering the behavior of bnAb combinations early in planning stages.

## Materials and Methods

### Study design

This was a non-randomized laboratory study designed to investigate the breadth and potency of HIV-1 bnAbs against a panel of 200 clade C HIV-1 Env pseudoviruses, and to develop mathematical models to predict combinations of 2, 3, or 4 bnAbs that would exhibit enhanced breadth, potency, extent of complete neutralization, and IIP relative to single bnAbs. Fifteen recently described bnAbs targeting four distinct epitopes on HIV-1 Env were each tested against the panel pseudoviruses *in vitro* to determine IC_50_ and IC_80_ titers and MPI. All neutralization assays were performed in duplicate and without blinding.

### Neutralization assays

Neutralizing antibody titers of bnAbs were determined using a luciferase-based assay in TZM.bl cells (NIH AIDS Research and Reference Reagent Program) as previously described [79, 80]. Unless stated otherwise, starting concentrations of individual bnAbs ranged from 10–50 μg/ml depending on the available supply at the time of testing. BnAbs were serially diluted seven times using 5-fold titration series. The concentration range tested for each bnAb is indicated in Table A in S1 Text. All assays were performed in a laboratory meeting GCLP standards.

### Viruses

A panel of 200 clade C HIV-1 Env pseudoviruses was utilized to assess the potency and breadth of bnAb neutralization activity. Functional Env clones were derived from individuals in acute/early stages of infection from South Africa (65%), Tanzania (14%), Malawi (11.5%), Zambia (6.5%), and Botswana (3%) collected over 12 years (1998–2010). All Envs were from heterosexual transmissions except for a single case of breastfeeding transmission. The majority of Envs exhibit a Tier 2 neutralization phenotype (75%, n = 150), with 1% classified as Tier 1A, 8.5% classified as Tier 1B, and 15.5% classified as Tier 3 [81]. Pseudovirus stocks were generated via transfection in 293T/17 cells (ATCC, Manassas, VA) and titrated using TZM.bl cells as previously described [82].

### Antibodies

A panel of 15 particularly broad and potent human monoclonal antibodies was selected based on prior data from testing against large multiclade panels of HIV-1 pseudoviruses. In some cases we included somatic variants or newly engineered variants that exhibited enhanced activity over parental wildtype bnAbs (i.e. 10-1074V, VRC07-523, CAP256-VRC26.25). Importantly, we included bnAbs that are currently in human clinical trials (VRC01, 3BNC117, 10–1074) or are advanced candidates for clinical testing (PGT121, 10E8, PGDM1400, CAP256-VRC26.25). Antibodies were generated in the laboratories of D. Burton at The Scripps Research Institute (PGT145, PGMD1400, PG9, PGT121, PGT128), M. Nussenzweig at The Rockefeller University (10–1074, 10-1074V, 3BNC117), or the NIH Vaccine Research Center (CAP256-VRC26.08, CAP256-VRC26.25, VRC01, VRC07, VRC07-523, VRC13, 10E8). VRC01 and VRC07 are CD4bs bnAbs of the same lineage [69]. VRC07-523 is an engineered clonal variant of VRC07 with increased potency and breadth [23]. Of note, VRC07-523 was made with a two amino acid mutation in the Fc domain (M428L/N424S) to increase affinity for the FcRn and therefore increase circulating *in vivo* half-life [83]; these mutations do not affect antibody-mediated neutralization. VRC13 is a CD4bs antibody that is distinct from the VRC01-class of antibodies in that it contacts gp120 primarily via CDR binding loops [70]. 10–1074 and PGT121 are clonal variants from the same donor [17]. 10-1074V is a variant of parental 10–1074 in which six complex-type glycan-contacting residues in IgH have been substituted with those from bnAb PGT121.

### Additive and Bliss-Hill models for predicting bnAb combination neutralization scores

For theoretical derivations of models, see S1 Text. The additive model [60] predicts combination IC_80_ as ${IC}_{80_{comb}}=1/(1/{IC}_{80_{A}}+1/{IC}_{80_{B}}+\ldots )$, where ${IC}_{80_{A}},{IC}_{80_{B}},\ldots$ are the single bnAb scores. The equation for combination IC_50_ is similar using single bnAb IC_50._

The Bliss-Hill model involves estimating single bnAb neutralization curves using Hill functions, *f*(*c*) = *c* ^*m*^/(*k* ^*m*^ + *c* ^*m*^), where *c* = bnAb concentration, *k* = *IC*_*50*_, and *m* = log(4)/[log(*IC*_*80*_)–log(*IC*_*50*_)]. The combination neutralization, using the Bliss Independence model, is *f* = 1 − (1 − *f*_*A*_)(1 − *f*_*B*_)(1 − *f*_*C*_) … where *f*_*A*_(*c*),*f*_*B*_(*c*),*f*_*C*_(*c*), … are the single bnAb neutralization functions and *c* is the bnAb concentration. This equation is solved for the combination IC_50_/IC_80_ (we use Brent algorithm[84, 85] implemented in Scipy [86]). Treatment of single bnAb IC_50_/IC_80_ values above or below experimental thresholds is detailed in the S1 Text.

For combinations with multiple bnAbs targeting the same epitope, the combined neutralization function of such bnAbs is calculated using $f_{A}(c)=(g_{A_{1}}(c)+g_{A_{2}}(c)+\ldots )/({1+g}_{A_{1}}(c)+g_{A_{2}}(c)+\ldots )$, where $g_{A_{i}}(c)=f_{A_{i}}(c)/\left(1-f_{A_{i}}(c)\right)$ and $f_{A_{i}}(c)$are Hill curves for each of the bnAbs *A*_*1*_, *A*_*2*_, … The Bliss independence model equation is used with the neutralization functions *f*_*A*_(*c*),*f*_*B*_(*c*),*f*_*C*_(*c*), … for each epitope to get neutralization by the combination.

### MPI predictions for bnAb combinations

Given the experimental or predicted MPI values for single bnAbs at a given concentration, *f*_*A*_,*f*_*B*_,*f*_*C*_,…, the combination MPI value was predicted as *f* = 1 − (1 − *f*_*A*_)(1 − *f*_*B*_)(1 − *f*_*C*_) …

### IIP predictions for bnAb combinations

IIP is defined as *IIP* = −*Log*_10_(1 − *f*(*c*)), where *f*(*c*) is the neutralization by a single bnAb or bnAb combination at concentration *c*. The Hill functions for neutralization by single bnAbs were calculated from IC_50_ and IC_80_ values, or by fitting experimental neutralization curves (S1 Text). For IIP of combinations, best-fit single bnAb neutralization functions together with BH model were used.

### Statistical analyses

All statistical analyses were performed using the Stats module in Scipy [86]. Non-parametric tests were preferred and two sided p-values are reported. False discovery rates (q-values) were calculated by using qvalue package for Python (https://github.com/nfusi/qvalue), based on the calculations outlined in reference [87].

## Supporting Information

S1 Text Supplementary Materials and Methods, figures, and tables. (DOCX)
