# Supplementary material for: Optimal Combinations of Broadly Neutralizing Antibodies for Prevention and Treatment of HIV-1 Clade C Infection
Source: PLoS Pathog. 2016 Mar 30;12(3):e1005520. doi: 10.1371/journal.ppat.1005520 (PMC4814126; doi:10.1371/journal.ppat.1005520)
Supplement: S1 Text — (DOCX) [file ppat.1005520.s001.docx]

**Supporting Information**

**Optimal Combinations of Broadly Neutralizing Antibodies for Prevention and Treatment of HIV-1 Clade C Infection**

Kshitij Wagh^1^, Tanmoy Bhattacharya^1, 2^, Carolyn Williamson^3^, Alex Robles^4^, Madeleine Bayne^4^, Jetta Garrity^4^, Michael Rist^4^, Cecilia Rademeyer^3^, Hyejin Yoon^1^, Alan Lapedes^1,^ Hongmei Gao^5^, Kelli Greene^5^, Mark K. Louder^6^, Rui Kong^6^, Salim Abdool Karim^7, 8^, Dennis R. Burton^9^, Dan H. Barouch^4^, Michel C. Nussenzweig^10^, John R. Mascola^6^, Lynn Morris^8, 11^, David C. Montefiori^5^, Bette Korber^1^, Michael S. Seaman^4^*

^1^ Theoretical Division, Los Alamos National Laboratory, Los Alamos, New Mexico, USA.

^2^ Santa Fe Institute, Santa Fe, New Mexico, USA.

^3^ Division of Medical Virology & Institute of Infectious Diseases and Molecular Medicine, University of Cape Town and NHLS, Cape Town South Africa.

^4^ Center for Virology and Vaccine Research, Beth Israel Deaconess Medical Center, Boston, Massachusetts, USA.

^5^ Department of Surgery, Duke University Medical Center, Durham, North Carolina, USA.

^6^ Vaccine Research Center, NIAID, NIH, Bethesda, Maryland, USA.

^7^ University of KwaZulu-Natal, Durban Department of Immunology and Microbial Science, Durban, South Africa.

^8^ Centre for the AIDS Programme of Research in South Africa (CAPRISA), University of KwaZulu-Natal, Durban, South Africa

^9^ The Scripps Research Institute, La Jolla, California, USA.

^10^ Laboratory of Molecular Immunology, The Rockefeller University, New York, New York, USA.

^11^ National Institute for Communicable Diseases (NICD), NHLS & University of the Witwatersrand, Johannesburg, South Africa.

**Supplementary Materials and Methods**

**Figures A-P**

**Tables A-B**

**Materials and Methods:**

**Theoretical Derivations of models using mass action kinetics**

***Single Antibody Neutralization***

The interactions between virions and antibody molecules were described using equilibrium mass action kinetics, briefly reviewed here in the context of antibodies/virions. The “chemical reaction” in question is (stoichiometry dropped for simplicity)

$V+A\rightleftarrows VA$

where V denotes virions and A denotes antibodies. Using mass action kinetics we get

Forward reaction rate $=k_{on}\left[ V \right]{[A]}^{s}$

Backward reaction rate $= k_{off}[VA]$

where [*V*] is concentration of unbound virions, [*A*] is the concentration of unbound antibodies, [*VA*] is the concentration of virion-antibody complexes, *k_on_* and *k_off_* are rate parameters and s is stoichiometry of neutralization, i.e. average number of antibody molecules required to neutralize one virion. The chemical equilibrium is reached when the forward and backward reaction rates are equal, and this relates the equilibrium concentrations as

$$\left[ VA \right]=k\left[ V \right]{[A]}^{s}$$

where, *k* = *k_on_ / k_off_* is the dissociation constant. To solve this equation, we note that [*V*] + [*VA*] = [*V_tot_*], the total concentration of virions. Thus, if *f* is the fraction of neutralized virions, then [*VA*] = *f* [*V_tot_*], and [*V*] = (1- *f*) [*V_tot_*]. If there is an excess of antibodies present in the assay, then [*A*] ≈ *c*, the concentration of antibodies in the assay. Using these in the above equation gives

$$f= \frac{k c^{s}}{1+k c^{s}}$$

which, can be reformulated in terms of IC_50_ (= *k*^1/s^) as

$$f= \frac{c^{s}}{{IC}_{50}^{s}+ c^{s}}$$

The following reformulation of the above equation will be used below

$$k c^{s}= \frac{f}{1-f}= \frac{c^{s}}{{IC}_{50}^{s}} (1)$$

We used the IC_50_ and IC_80_ values for a given bnAb and virus to calculate *s* using the formula $s = log(4) / (log({IC}_{80}) - log({IC}_{50}))$. Using this and IC_50_ values, we were able to estimate the neutralization curves for each bnAb versus each virus.

The parameter *s* is called slope [71] or Hill coefficient and can be interpreted as the stoichiometry of the “chemical reaction” in question. The stoichiometry of neutralization is not known. While the Bliss-Hill (BH) model works with arbitrary *s*, the Additive model requires that we assume *s* ≈ 1. This assumption is largely valid for CD4bs and V3-glycan bnAbs, but is an overestimate for V2-glycan and MPER bnAbs [65, 71, 72].

Of note, the assumption *s* ≈ 1 suggests that 1 antibody molecule is required to neutralize one virion, but on an average a virion has ~10 Env trimers on its surface. The value of *s* in the equation above, however, is an “effective” stoichiometry of neutralization, which reproduces the average behavior seen in experimental assays. The underlying kinetics of antibody-virion neutralization is more complicated than the simple model outlined above. In ref. [88], it has been modeled as follows. If antibodies are bound to more than a minimum threshold of the available epitopes within a trimer, then the trimer is deactivated, and if more than a threshold number of trimers on a virion are deactivated then that virion is neutralized. This modeling is impacted by factors like within-clonal-population heterogeneity in the total number of Env trimers on virions, epigenetic variation in the forms of expressed Envs (such as distinct carbohydrate additions) that result in variation of the rate constants for antibody binding to a trimer, etc. Our preliminary modeling analyses indicate that these factors can give reduced effective *s* values in the range of 1.

***Bliss-Hill Model for neutralization by antibody combinations****.*

The modeling of neutralization by antibody combinations needs to account for the different configurations of virion-antibody complexes. So for example for 2-bnAb combinations, with bnAbs A and B for example, we can now have virions bound with either A or B, and also virions simultaneously bound with A and B. Thus, the chemical reactions in this case would be (stoichiometry dropped for simplicity)

$$V+A\rightleftarrows VA$$

$$V+B\rightleftarrows VB$$

$$VA+B\rightleftarrows VAB\rightleftarrows VB+A$$

Using mass action kinetics and the assumption of independent binding of antibodies to virions (same rate constants of binding for a given bnAb, with or without the other bnAb bound to a virion), we find, by requiring the forward and backward reaction rates to be equal in equilibrium, that

$$\left[ VA \right]= k_{A}\left[ V \right]{[A]}^{s_{A}}$$

$$\left[ VB \right]= k_{B}\left[ V \right]{[B]}^{s_{B}}$$

$$\left[ VAB \right]= k_{A}\left[ VB \right]{[A]}^{s_{A}}= k_{B}\left[ VA \right]{[B]}^{s_{B}}= k_{A}k_{B}[V]{[A]}^{s_{A}}{[B]}^{s_{B}}$$

where [VA] and [VB] are the concentrations of virions bound by A and B, respectively, and [VAB] is the concentration of virions simultaneously bound by both A and B, s_A_ and s_B_ are the stoichiometric factors for A and B, respectively, and k_A_ and k_B_ are the corresponding rate constants. In the last equation, we have neglected the term for simultaneous binding of an unbound virion by both A and B molecules, and further assumed that neither the rate constants nor the stoichiometric factors for A and B binding depend on whether the other antibody has already bound. In general, we can show that similar assumptions imply

$$\left[ VABCD\ldots\right]= k_{A}k_{B}k_{C}k_{D}\ldots{\left[ V \right]\left[ A \right]}^{s_{A}}\left[ B \right]^{s_{B}}\left[ C \right]^{s_{C}}\left[ D \right]^{s_{D}}\ldots(2)$$

Using the assumption that any virion bound to at least one bnAb is neutralized, the fraction of virions neutralized for the 2-bnAb combination case is

$$f= \frac{\left[ VA \right]+\left[ VB \right]+[VAB]}{\left[ V \right]+\left[ VA \right]+\left[ VB \right]+[VAB]}$$

Using equation (2), we get

$$f= \frac{k_{A}c^{s_{A}}+k_{B}c^{s_{B}}+k_{A}k_{B}c^{s_{A}}c^{s_{B}}}{{1+k}_{A}c^{s_{A}}+k_{B}c^{s_{B}}+k_{A}k_{B}c^{s_{A}}c^{s_{B}}}$$

which can rewritten as

$$f= \frac{\left( 1+k_{A}c^{s_{A}} \right)\left( 1+k_{B}c^{s_{B}} \right)-1}{\left( 1+k_{A}c^{s_{A}} \right)\left( 1+k_{B}c^{s_{B}} \right)}$$

This equation can be generalized for any number of antibodies in the combination as

$$f= \frac{\left( 1+k_{A}c^{s_{A}} \right)\left( 1+k_{B}c^{s_{B}} \right)\left( 1+k_{C}c^{s_{C}} \right)\left( 1+k_{D}c^{s_{D}} \right)\ldots-1}{\left( 1+k_{A}c^{s_{A}} \right)\left( 1+k_{B}c^{s_{B}} \right)\left( 1+k_{C}c^{s_{C}} \right)\left( 1+k_{D}c^{s_{D}} \right)\ldots} (3)$$

The expansion of the product in the numerator accounts for all possible configurations of virions bound with 2, 3, 4… antibodies using equation (2) (for example for A and B simultaneously bound, $\left[ VAB \right]= k_{A}k_{B}\left[ V \right]c^{s_{A}}c^{s_{B}}$). Using equation (1), we can relate $k_{A}c^{s_{A}}, k_{B}c^{s_{B}}, \ldots$to the single antibody neutralization curves *f_A_*(*c*), *f_B_*(*c*), …

$$f= \frac{\left( 1+ \frac{f_{A}}{1-f_{A}} \right)\left( 1+ \frac{f_{B}}{1-f_{B}} \right)\left( 1+ \frac{f_{C}}{1-f_{C}} \right)\left( 1+ \frac{f_{D}}{1-f_{D}} \right)\ldots-1}{\left( 1+ \frac{f_{A}}{1-f_{A}} \right)\left( 1+ \frac{f_{B}}{1-f_{B}} \right)\left( 1+ \frac{f_{C}}{1-f_{C}} \right)\left( 1+ \frac{f_{D}}{1-f_{D}} \right)\ldots}$$

which gives,

$$f=1-\left( 1-f_{A} \right)\left( 1-f_{B} \right)\left( 1-f_{C} \right)\left( 1-f_{D} \right)\ldots(4)$$

This equation can be understood easily: under the assumption of independent binding, the fraction of virions not bound by any antibody is given by $\left( 1-f_{A}(c) \right)\left( 1-f_{B}(c) \right)\ldots$, and so the the total fraction neutralized by a combination is one minus the fraction of virions not bound by any bnAb. This equation gives the Bliss-Hill model equations for neutralization by combinations. This general equation can be rewritten for the specific cases of 2-, 3-, and 4- bnAb combinations as:

$f(c)= f_{A}\left( c \right)+\left( 1-f_{A}\left( c \right) \right)f_{B}(c)$, for 2-bnAb combinations

$f\left( c \right)= f_{A}\left( c \right)+\left( 1-f_{A}\left( c \right) \right)f_{B}+\left( 1-f_{A}\left( c \right) \right)\left( 1-f_{B}\left( c \right) \right)f_{c}(c)$, for 3-bnAb combinations, and for 4-bnAb combinations

$f\left( c \right)= f_{A}\left( c \right)+\left( 1-f_{A}\left( c \right) \right)f_{B}+\left( 1-f_{A}\left( c \right) \right)\left( 1-f_{B}\left( c \right) \right)f_{c}\left( c \right)+\left( 1-f_{A}\left( c \right) \right)\left( 1-f_{B}\left( c \right) \right)\left( 1-f_{C}\left( c \right) \right)f_{D}(c)$ (5).

Several of the combinations considered had multiple bnAbs targeting similar epitopes. The treatment for such situations is different, due to the fact there will be increased steric hindrance between similar specificity bnAbs. To account for this, we considered the extreme scenario that the concentration of doubly-bound virions, i.e. [*VA_1_A_2_*] = 0 for combination with bnAbs *A_1_* and *A_2_* targeting the same epitope. Steric hindrance usually will not be this severe, since a given virion has multiple epitopes of a given type. In order to capture the effect of multiple binding sites, we would need a more detailed model of antibody-virion interactions, which accounts for the total number of Env trimers on a virion, the binding of bnAbs to a single Env trimer, the single trimer deactivation stoichiometry, and the virion deactivation stoichiometry [88]. Too little is currently known about the needed parameters to enable the use of such a high parameter model. We, therefore, prefer the simple model using the number of virions and the simplifying assumption that multiple bnAbs of the same specificity cannot bind a single virion. We note that even though a strict justification is lacking, the observed effective stoichiometries in all our models turn out to be close to 1 instead of the number of binding sites on the virion. Implementation of our approach yields better predictions than the regular BH model for the IC_80_ values for two 2-bnAb combinations with repeat specificity, CAP256-VRC26.25 + PGDM1400 and 10-1074V + PGT128 (Fig. B).

The assumption of complete steric hindrance implies that no configurations of virions bound by multiple same-specificity bnAbs, such as [*VA_1_A_2_*], [*VA_1_A_2_B*], [*VA_1_A_2_BC*], etc, are allowed. To formulate these constraints, we note that in equation (3), the product of the terms from different brackets account for the configurations of simultaneously bound virions. Thus, if we consider the following

$$f= \frac{\left( 1+k_{A_{1}}c^{s_{A_{1}}}+k_{A_{2}}c^{s_{A_{2}}} \right)\left( 1+k_{B}c^{s_{B}} \right)\left( 1+k_{C}c^{s_{C}} \right)\ldots-1}{\left( 1+k_{A_{1}}c^{s_{A_{1}}}+k_{A_{2}}c^{s_{A_{2}}} \right)\left( 1+k_{B}c^{s_{B}} \right)\left( 1+k_{C}c^{s_{C}} \right)\ldots}$$

we ensure that we do not get any products that have terms for both A_1_ and A_2_, and account for all other allowed configurations. Using equation (1), we get

$$f= \frac{\left( 1+ \frac{f_{A_{1}}}{1-f_{A_{1}}}+\frac{f_{A_{2}}}{1-f_{A_{2}}} \right)\left( 1+ \frac{f_{B}}{1-f_{B}} \right)\left( 1+ \frac{f_{C}}{1-f_{C}} \right)\ldots-1}{\left( 1+ \frac{f_{A_{1}}}{1-f_{A_{1}}}+\frac{f_{A_{2}}}{1-f_{A_{2}}} \right)\left( 1+ \frac{f_{B}}{1-f_{B}} \right)\left( 1+ \frac{f_{C}}{1-f_{C}} \right)}$$

Using the definition

$$g_{A}=\frac{\frac{f_{A_{1}}}{1-f_{A_{1}}}+\frac{f_{A_{2}}}{1-f_{A_{2}}}+\ldots}{1+\frac{f_{A_{1}}}{1-f_{A_{1}}}+\frac{f_{A_{2}}}{1-f_{A_{2}}}+\ldots} (6)$$

we can rewrite *f* as

$$f= \frac{\left( 1+\frac{g_{A}}{1-g_{A}} \right)\left( 1+\frac{f_{B}}{1-f_{B}} \right)\left( 1+\frac{f_{C}}{1-f_{C}} \right)\ldots-1}{\left( 1+ \frac{g_{A}}{1-g_{A}} \right)\left( 1+\frac{f_{B}}{1-f_{B}} \right)\left( 1+\frac{f_{C}}{1-f_{C}} \right)}$$

$$=1-\left( 1-g_{A} \right)\left( 1-f_{B} \right)\left( 1-f_{C} \right)\ldots$$

Thus, the difference in formulation for neutralization by combinations with similar specificity bnAbs amounts to first calculating the functions $g_{A}\left( c \right),g_{B}\left( c \right),\ldots$ for each epitope by combining the single bnAb neutralization functions for bnAbs targeting the same specificity by using equation (6), and then using the BH equations (equation 5) with $g_{A}\left( c \right),g_{B}\left( c \right),\ldots$to get the neutralization function for the combination. It should be noted that for a single bnAb, equation (6) gives$g_{A}\left( c \right)= f_{A}(c)$. Thus, for any (non-zero) number of bnAbs in the combination targeting a given specificity, we can condense the entire formalism as always using the equation

$$f=1-\left( 1-g_{A} \right)\left( 1-g_{B} \right)\left( 1-g_{C} \right)\left( 1-g_{D} \right)\ldots$$

with the factors $g_{A}\left( c \right),g_{B}\left( c \right),\ldots$ obtained by combining the fractions of viruses bound by individual antibodies targeting the same or sterically hindering epitopes using equation (6).

The above equations were developed in the context of enzyme kinetics [89]. To our knowledge, this is the first work to extend these ideas for the prediction of antibody combination neutralization, IC_50_/IC_80_ titers as well as full curves, from single antibody neutralization scores. Verrier et al. [90] also used Bliss independence model for analyzing neutralization by combination of antibodies, however they used experimental single antibody neutralization curves and did not use the parameterize the single antibody curves using Hill functions, as done here.

***Derivation of Additive Model using mass action kinetics***

The additive model was used in Kong et al. [60], to explain the combination neutralization scores in terms of the single bnAb neutralization scores. While the agreement between observed titers and additive model predictions was found to be quite good, the BH model above improved the predictions significantly, and thus, we favor the use of the BH model (Fig. 2 and Fig. C). When both models were applied to the C clade data set to predict the behavior of bnAb combinations for single antibody data, the additive model predictions led to the same choice of the best-in-category combinations and similar relative trends in potency-breadth profiles (Fig. I). However, some differences in ordering of best-in-category 3 and 4 bnAb combinations with very similar geometric mean IC_80_ were found.

This section describes the theory behind the additive model, and outlines the assumptions that go into its formulation, to enable a comparison with the BH model and provide context regarding our original use of the additive model in Kong et al. [60]. The single antibody neutralization curve can be obtained by using mass action kinetics as detailed above. If we *assume the effective stoichiometry of neutralization is 1*, then we obtain the neutralization curve for a single antibody as

$$f= \frac{c}{c+ {IC}_{50}}$$

A useful form of this equation is

$$\frac{c}{{IC}_{50}}= \frac{f}{1-f} (7)$$

For neutralization by combinations, we had seen that (equation 4)

$$f= \frac{\left( 1+ \frac{f_{A}}{1-f_{A}} \right)\left( 1+ \frac{f_{B}}{1-f_{B}} \right)\left( 1+ \frac{f_{C}}{1-f_{C}} \right)\left( 1+ \frac{f_{D}}{1-f_{D}} \right)\ldots-1}{\left( 1+ \frac{f_{A}}{1-f_{A}} \right)\left( 1+ \frac{f_{B}}{1-f_{B}} \right)\left( 1+ \frac{f_{C}}{1-f_{C}} \right)\left( 1+ \frac{f_{D}}{1-f_{D}} \right)\ldots}$$

In this equation the product terms (e.g. [*f_A_*/(1-*f_A_*)] [*f_B_*/(1-*f_B_*)]) arise from the terms describing the virions bound by multiple bnAbs simultaneously (e.g. [*VAB*]). The additive model is obtained when the *concentration of such simultaneously bound virions is negligible compared to the concentrations of virions bound by only one antibody*.

How valid is this assumption? Intuitively, when multiple antibodies in a combination all bind a large fraction of a given virus, then, assuming independent binding, the concentration of simultaneously bound virions should be high. For example, if two antibodies A and B both have an IC_50_=1µg/ml, then at concentration 1µg/ml of each, *f*/(1-*f*) = 1 for both of them, and the product [*f_A_*/(1-*f_A_*)] [*f_B_*/(1-*f_B_*)] = 1 as well, and not negligible. On the other hand, if one antibody in the combination has much higher potency than the rest, then at equal concentrations of the antibodies, the fraction of multiply bound virions will be low and most of the virions will be bound by the higher potency antibody (in our effective modeling approach, potency and binding are completely equivalent). The neutralization profiles of V2-glycan and V3-glycan bnAbs are complementary (Fig. 1, E-F), and when they neutralize a virus, these are more potent than CD4bs bnAbs, which in turn are more potent than 10E8. Thus, we find that for many viruses one bnAb that is more potent than the rest, leading to the approximate validity of the additive model.

Under this condition, we obtain

$$f_{add}= \frac{\frac{f_{A}}{1-f_{A}}+\frac{f_{B}}{1-f_{B}}+\frac{f_{C}}{1-f_{C}}+\frac{f_{D}}{1-f_{D}}+\ldots}{1+\frac{f_{A}}{1-f_{A}}+\frac{f_{B}}{1-f_{B}}+\frac{f_{C}}{1-f_{C}}+\frac{f_{D}}{1-f_{D}}+\ldots}$$

where, *f_add_* is the neutralization by the combination under the additive model and *f_A_*, *f_B_*, *f_C_*, *f_D_,…* are the single bnAb neutralization functions. This equation can be rewritten as

$$\frac{f_{add}}{1-f_{add}}= \frac{f_{A}}{1-f_{A}}+\frac{f_{B}}{1-f_{B}}+\frac{f_{C}}{1-f_{C}}+\frac{f_{D}}{1-f_{D}}+\ldots(8)$$

By using equation (7), we then get

$$\frac{f_{add}}{1-f_{add}}= \frac{c}{{IC}_{{50}_{A}}}+\frac{c}{{IC}_{{50}_{B}}}+\frac{c}{{IC}_{{50}_{C}}}+\frac{c}{{IC}_{{50}_{D}}}+\ldots(9)$$

where c is the concentration of each bnAb in the combination. The combination IC_50_ $\left( {IC}_{{50}_{comb}} \right)$can be found by setting *f_add_* = 0.5 in equation (9), which gives

$$1= \frac{{IC}_{{50}_{comb}}}{{IC}_{{50}_{A}}}+\frac{{IC}_{{50}_{comb}}}{{IC}_{{50}_{B}}}+\frac{{IC}_{{50}_{comb}}}{{IC}_{{50}_{C}}}+\frac{{IC}_{{50}_{comb}}}{{IC}_{{50}_{D}}}+\ldots$$

or,

$$\frac{1}{{IC}_{{50}_{comb}}}= \frac{1}{{IC}_{{50}_{A}}}+\frac{1}{{IC}_{{50}_{B}}}+\frac{1}{{IC}_{{50}_{C}}}+\frac{1}{{IC}_{{50}_{D}}}+\ldots(10)$$

Similarly, to obtain combination IC_80_, we set *f_add_* = 0.8 in equation (9), and use the relation IC_80_ = 4 IC_50_ which follows from equation (7) (this is the implication of the assumption of slope/stoichiometry of 1). This gives

$$\frac{1}{{IC}_{{80}_{comb}}}= \frac{1}{{IC}_{{80}_{A}}}+\frac{1}{{IC}_{{80}_{B}}}+\frac{1}{{IC}_{{80}_{C}}}+\frac{1}{{IC}_{{80}_{D}}}+\ldots(11)$$

Equations (10-11) are the formulations for the additive model. In general, the form of these equations is the same for arbitrary neutralization (and not just 50 and 80% that we have considered), which follows from equations (7-8).

***Derivation of Additive Model*** ***using Loewe’s method***

The form of the additive model (equations 10-11) has a long history. The earliest argument for it is commonly attributed to Loewe [91-94] in the context of combining different drugs that have similar action. This early argument is not based on the principles of mass action used above, and is often used in other studies on combinations of reagents [95], so we summarize it below.

Suppose two different reagents, A and B, individually produce similar effects. In the context of HIV-1 infection the reagents may be two different monoclonal antibodies that inhibit infection. In other contexts the reagents may be e.g. two different drugs affecting pain control such as acetaminophen and tramadol [96]. Suppose reagent A has ${IC}_{{50}_{A}}$ = 2mg/ml and reagent B has ${IC}_{{50}_{B}}$= 5mg/ml (the following argument works for any effect level). Thus, 5 mg/ml of B is therefore the “dose equivalent” of 2 mg/ml of A at the 50% effect level. The potency ratio, defined as $R={{IC}_{{50}_{B}}}/{{IC}_{{50}_{A}}}$, is thus 5/2 or 2.5 at the 50% effect level in this example. If we assume that the potency ratio, e.g. 2.5, is *constant at all effect levels* including 20%, 50%, 80%, etc., then any dose “*a*” of reagent A producing some effect has an equivalent effective dose, “$b_{equiv}$”, of reagent B producing the same effect, where $b_{equiv}=R a= \left( {{IC}_{{50}_{B}}}/{{IC}_{{50}_{A}}} \right) a$. Consider a combination of A and B, where A and B are present in concentrations “a” and “b” respectively, and that at these concentrations this combination achieves 50% effect. Given the assumption of equivalent effective dose, the replacement of dose “*a*” of A in the combination with an equivalent dose of B, $b_{equiv}$, will have the same effect as the original combination of A and B. Thus, the combination of A and B will be equivalent to having B alone at the dose of $b+b_{equiv}=b+a\left( {{IC}_{{50}_{B}}}/{{IC}_{{50}_{A}}} \right)$. Since the combination of A and B has 50% effect, this new dose of B alone also has a 50% effect and therefore,

$$b+ \frac{{IC}_{{50}_{B}}}{{IC}_{{50}_{A}}}a={IC}_{{50}_{B}}$$

Dividing by ${IC}_{{50}_{B}}$ gives

$$\frac{b}{{IC}_{{50}_{B}}}+ \frac{a}{{IC}_{{50}_{A}}}=1$$

This expression gives the mass action derived additive model equation (10) by assuming that antibodies in the combination have same concentration and this concentration is defined as the concentration of the combination, i.e. $a=b={IC}_{{50}_{comb}}$. It gives a simple additive (linear) relation between values of dose *a* and dose *b* that produce a 50% effect, given the assumptions, which is the concept behind the “isobologram” (see ref. [91] for a review), defined as the locus of boluses of A and B that produce the same effect. The expression $a/{{IC}_{{50}_{A}}}+b/{{IC}_{{50}_{B}}}$ is the “combination index” (CI) [92] and deviations of CI from the value 1 have been used to define synergy (more than 1) and inhibition (less than 1).

The result of the argument above (as well as arguments based on mass action) is subject to the validity of the underlying assumptions. As discussed by Grabovsky and Tallarida [94], the above assumption of constant dose equivalence at all effect levels requires that each reagent have identical slopes (*s* in equation (1)). If the slopes are different then simple algebra shows that dose equivalence is not constant at all effect levels. This assumption limits the applicability of this approach to antibody combinations, due to different classes of bnAbs having different slopes [71, 72].

Since the dose equivalence assumption is satisfied for antibodies with equal slopes, and the additive model follows from this assumption as outlined above, a natural question arises – would the additive model always be applicable for such cases? We have shown that this is not always true, and depends on the choice of model used. If we use mass action kinetics with independent binding (the BH model derivation above), we do not get the additive model even if the individual antibody slopes are equal to each other and even if they are equal to 1. To obtain the additive model using mass action kinetics (see above), we need to assume complete steric hindrance (no virions are simultaneously bound by antibodies A & B, say). This can be seen by using equation (8) together with equation (1), and assuming that each bnAb has the same concentration *c* and same slope *s*, which gives the generalization of equation (9) to arbitrary slopes

$$\frac{f_{add}}{1-f_{add}}= \left( \frac{c}{{IC}_{{50}_{A}}} \right)^{s}+\left( \frac{c}{{IC}_{{50}_{B}}} \right)^{s}\ldots$$

This equation gives the combination ${IC}_{50}$ as

$$\left( \frac{1}{{IC}_{{50}_{Comb}}} \right)^{s}= \left( \frac{1}{{IC}_{{50}_{A}}} \right)^{s}+\left( \frac{1}{{IC}_{{50}_{B}}} \right)^{s}\ldots$$

This expression reduces to the additive model only for *s = 1*.

Thus, the Loewe argument is consistent with mass action modeling only if we assume complete steric hindrance (or mutual exclusivity as in ref. [89]) and individual reagent slopes equal to 1. This suggests that the Loewe argument is not completely model-independent and requires additional justification to assert that the combination of reagents behaves as a single reagent at an effective higher concentration. It is unclear what that justification would be in the case of combinations of different antibodies.

**Treatment of threshold IC_50_ and IC_80_ values for estimating single bnAb Hill curves.**

We used single bnAb IC_50_ and IC_80_ values to estimate the full neutralization curves assumed to be Hill curves, f(c) = c^s^/(k^s^ + c^s^), where c is concentration, k = IC_50_ and s = log(4)/(log(IC_80_) – log(IC_50_)). For a given virus, if any of the antibodies in a bnAb combination had both IC_50_ and IC_80_ above the highest concentration threshold, then the contribution of such antibodies was neglected for the combination neutralization for this virus. For an antibody in a combination if both IC_50_ and IC_80_ were below the lowest concentration threshold, then the combination IC_50_ and IC_80_ were reported at the lowest threshold concentration. If only one of the bnAb IC_50_ or IC_80_ was either below (above) the experimental concentration thresholds, then the corresponding IC value was set at the lower (higher) concentration threshold.

**Modeling Systematic Deviations from Bliss-Hill Model predictions**

Despite the improved prediction accuracy, the experimental data for bnAb combinations still showed residual systematic deviations from BH model, similar in direction for some antibody classes to those previously observed by using the additive model [60]. In particular, we found several viruses for which the combination was less potent than the most potent bnAb in the combination (note that for each virus, both additive and BH models predict that the combination is more potent than the most potent component bnAb). This effect was most evident for combinations with CAP256-VRC26.25, where the observed combination IC_80_ titer was 3-fold or higher than the IC80 titer for CAP256-VRC26.25 (IC_80_ = 0.0003 μg/ml) for 2-5 viruses (out 20 total) (Fig. D, points where both the additive and BH model predictions are at 0.0003 μg/ml and observed titers in the range 0.001-0.01 μg/ml). Such potentially inhibitory cases for a few viruses were also found in Kong et al. [60]. The deviations from BH model for most combinations were in the same direction as the deviations from additive model, however for some combinations, the direction of deviations were different for both models (combinations ‘abc’ in Fig. D and 'abc', 'bcd', 'abcd' in Fig. E). We analyzed the Kong et al. data to model the patterns of deviations from the BH predictions, with a view to use this “deviation modeling” to improve the predictions over those from the BH model.

We used several candidate models with 1–4 parameters to see if we could improve the fits for the deviations from BH model for each bnAb combination. For bnAb combinations, deviations were defined as Log_10_(Predicted IC_80_) – Log_10_(Observed IC_80_), such that positive (negative) deviation implies more (less) potent neutralization than predicted. Motivated by the patterns in deviations seen for combinations from the Kong et al. dataset (Fig. F), we chose five candidate functions with BH-predicted combination Log_10_(IC_80_) as the independent variable for modeling : i. no deviation (y=0, null model), ii. a nonzero constant, iii. a linear function, iv. a two part continuous function made of a linear function and a constant (i.e., y = min(a x + b, c)), and v. a two-part continuous function made of two linear functions (i.e., y = min(a x + b, c x + d)). Best-fit parameters for each function were found using least squares optimization routine in SciPy [86]. We used Bayesian information criterion (BIC) to choose the model that best explained the deviation patterns, while penalizing higher-complexity models. For BIC calculations, to find the likelihood of the data given a model, we assumed identical independent Gaussian errors, with standard deviation estimated from the data itself. Since, the deviation models are a function of the BH-predicted Log_10_(IC_80_) scores, combinations having different overall potency will have different deviation models, even though they might have similar trends in deviations. Thus, to increase the predictive power of the deviation model learnt for one combination when applied to deviations for other combinations of the same class, we normalized the BH-predicted Log_10_(IC_80_) scores to have zero medians. This procedure does not impact the choice of the best-fit models because these were linear functions. Deviation modeling corrected IC_80_ predictions were calculated as ${IC}_{80}= {IC}_{{80}_{BH}} {10}^{-f({IC}_{{80}_{BH}})}$, where *f(x)* is the best-fit deviation model for a given combination and ${IC}_{{80}_{BH}}$ is the BH predicted IC_80_.

Using Bayesian Information Criterion (BIC), we found that for some combinations, using the IC_80_ data, the best fit was the BH model. These combinations were CD4bs+V2g, CD4bs+MPER and CD4bs+V2g+MPER, across both Kong et al. datasets, and CD4bs+V3g and CD4bs+MPER, in one of the two Kong et al datasets (Fig. F). However for other combinations, we found that we could fit the experimental data better by modeling deviations between observed and predicted Log_10_ IC_80_ values as a linear function of BH-predicted Log_10_ IC_80_. In these cases, we found that the deviations were such that experimentally tested combinations behaved less potently than predicted for more sensitive viruses, and more potently than predicted for the less sensitive viruses.

To test the predictive power of deviation modeling, we used the fact that Kong et al. panel comprises two datasets. These differ in utilizing different bnAbs targeting CD4bs and V3-glycan epitopes, but have the same bnAbs targeting V2-glycan and MPER epitopes. We applied the deviation model learned for a given combination in one dataset (e.g. VRC07 + PGT128), to the analogous combination from the other dataset (e.g. 3BNC117 + 10-1074), and assessed if this deviation modeling increased the accuracy of predictions. We found that for the combinations V2g+MPER, CD4bs+V2g+V3g and V2g+V3g+MPER, deviation modeling from the other dataset significantly improved predictions over the BH model (p < 0.023 and q < 0.036 using the paired Wilcoxon test on the Log_10_ deviations between observed and predicted IC_80_). However, for other combinations we did not see any consistent significant improvement. Although, the V2g+MPER combination was repeated in the Kong et al. datasets, the other two combinations that showed improved predictions using deviation modeling had one or two bnAbs that differed between the two datasets. This result suggests that for some bnAb combinations, deviation modeling learned from similar epitope targeting combinations from a different dataset could improve predictions over the BH model. Consistent with this, we found that deviation modeling learned from the Kong et al. datasets significantly improved predictions for the analogous clade C panel combinations of the type V2g+MPER (p = 0.03 using paired Wilcoxon test on Log_10_ IC_80_ deviations) (CD4bs+V2g+V3g also showed improved prediction, though it did not reach statistical significance by the Wilcoxon test) (Fig. G). The deviation models learnt from Kong et al. datasets were averaged to calculate the predictions for the clade C panel. We note that even though for some combinations the deviations from BH model have a systematic and predictable trend, and the deviation modeling can exploit this to improve prediction, the magnitude of these deviations are small in absolute terms and the predictions from BH model are quite accurate. Indeed, the deviation modeling to predict combination IC_80_ scores did not affect the main conclusions obtained using BH modeling (Fig. H).

**Simultaneous neutralization by multiple bnAbs**

The number of bnAbs in a combination actively neutralizing a virus was defined as the number of bnAbs with single bnAb IC_80_ less than a threshold. However, for combinations with multiple bnAbs targeting the same epitope, a modified counting procedure was employed to calculate the number of simultaneously active bnAbs. If any of the multiple specificity bnAbs in the combination neutralized a given virus at an IC_80_ threshold, then their contribution accounted for one bnAb active, and if none of them neutralized a given virus, then their contribution accounted for zero bnAbs active. The implication of this modified counting procedure is that even if multiple same-specificity bnAbs were active for a given virus, they will still be counted as a maximum of single bnAb active. This simplified counting scheme was guided by two related observations. First, the neutralization profiles show that there is considerable, but not complete, overlap in the viruses missed by bnAbs targeting the same epitope (Fig. 1E and F). Second, bnAbs targeting a similar specificity can have shared sensitivity/resistance-associated mutations (e.g. position 332 and 334 glycans for V3-glycan bnAbs, 160 glycan for V2-glycan bnAbs and 276 glycan for CD4 bnAbs [97]). These observations suggest that evolution of escape from one bnAb could also lead to escape from other bnAbs targeting the same epitope, and hence, the modified counting scheme was introduced to capture this reduced barrier for escape from multiple bnAbs targeting the same epitope.

For Fig. J, we used the single bnAb activity threshold of IC_80_ < 50 µg/ml and <100 µg/ml. These concentrations are beyond the highest tested concentrations, and thus for this analysis, the single bnAb neutralization curves were extrapolated using the modeling of single bnAb neutralization curves (see “*Curve fitting for experimental single bnAb neutralization curves”* below).

**Observed MPI for single bnAbs.**

The experimental MPI values shown in Fig. 6 for 10-1074V are based on assays with maximum bnAb concentration of 10 µg/ml (n=25) and 20 µg /ml (n=175), while for other bnAbs the assays had a maximum concentration of 25 µg /ml. Thus, for 10-1074V and its combinations, the values shown are underestimates of actual MPI values if the experiments went up to a concentration of 25 µg /ml. However, the estimated difference (using best-fit Hill curves, “Curve fitting for single bnAb neutralization curves” below) was found to be quite small for 10-1074V alone (predicted median error = 0.0%, 95% CI = 0-19% neutralization) and even smaller for combinations.

**Curve fitting for experimental single bnAb neutralization curves.**

Hill curves were used to fit single bnAb neutralization curves that have the form $f\left( c \right)= {c^{m}}/\left( c^{m}+ k^{m} \right)$, where *c* is bnAb concentration, *k* is IC_50_, and *m* is the Hill exponent or slope [73]. Due to the experimental uncertainties in measuring low neutralization, we chose to set all data points with neutralization <= 10% as 10%, and fit the points to the modified function f_mod_ (c) = max(10%, f (c)). This procedure uses the information about the onset in the curve fitting without being impacted by the actual values in this regime. The best-fit parameters were estimated using least squares optimization in Scipy.

For cases when full experimental curves were not available, the neutralization functions for single bnAbs were assumed to be Hill curves, with *k* = *IC_50_* and *m* = log(4)/ [log(*IC_80_*) – log (*IC_50_*) ].

**Error modeling for curve fitting of single bnAb neutralization curves.**

We modeled experimental single bnAb neutralization curves as Hill curves, to estimate incomplete neutralization and instantaneous inhibitory potential (IIP) of single bnAbs and bnAb combinations at high concentrations. To understand the impact of uncertainty in the experimental data on the conclusions from these analyses, we modeled the following types of errors:

1. *Experimental uncertainty in measuring neutralization.* The experimental assays were performed in replicates and the differences in neutralization responses for the replicate experiments for each measurement were used to model the errors. These replicate differences were found to be negatively correlated with average neutralization values, and we modeled the difference as linear function of neutralization (4.0% difference at 0% neutralization to 0.6% at 100% neutralization). This function was used as the standard deviation to generate random Gaussian errors (with zero mean) in neutralization measurements.
2. *Uncertainty in bnAb concentrations.* Motivated by the common observations of shifts in repeat neutralization curves (Fig. M), we modeled the uncertainty in concentration measurements as follows. With probability 0.5, all concentration points below a randomly chosen concentration point in the experimental curve were multiplied by Log-uniform random number between 0.2 and 5. We used two probability distributions to choose a concentration point on the experimental curve—a uniform distribution on the eight concentration points, or a linearly decreasing function on the rank of the concentration points with highest rank point corresponding to the lowest concentration. Both these approaches yielded similar uncertainty in quantities of interest calculations.

The errors from these error models were used to generate 1,000 artificial bootstrap replicates including error on each given experimental curve. The best-fit curves for each of these replicates were estimated as in Methods, and using these estimates, the quantities of interest (e.g. neutralization at 100 µg/ml) were calculated. The distributions of the quantities of interest were found to be close to Gaussian (data not shown).

**CombiNaber – a webtool for predicting combination neutralization scores**

A webtool, CombiNaber will be made available at the Los Alamos HIV Database (http://www.hiv.lanl.gov/content/sequence/ABCPRED/abcpred.html), which using single antibody IC_50_ and/or IC_80_ values as input, will predict the neutralization scores for all 2, 3 and 4 bnAb combinations. If only IC_50_ or IC_80_ values are given, the additive model will be used, and if both are given, the user will be able to choose between additive, BH or BH+deviation models (see “*Modeling systematic deviations from Bliss-Hill model predictions*” above) for predictions. For use of BH model or BH+deviation models, the user also will need to input the class of antibodies (V2 glycan, V3 glycan, CD4bs, etc.) with the possibility of giving a wildcard class such as for novel epitopes.

This tool will also systematically compare the potency-breadth profiles and coverage with multiple bnAbs active to provide the user with best combination candidates for their panel.

**Supporting References:**

88. Brandenberg OF, Magnus C, Regoes RR, Trkola A. The HIV-1 Entry Process: A Stoichiometric View. Trends Microbiol. 2015;23(12):763-74.

89. Chou TC, Talalay P. Generalized equations for the analysis of inhibitions of Michaelis-Menten and higher-order kinetic systems with two or more mutually exclusive and nonexclusive inhibitors. Eur J Biochem. 1981;115(1):207-16.

90. Verrier F, Nadas A, Gorny MK, Zolla-Pazner S. Additive effects characterize the interaction of antibodies involved in neutralization of the primary dualtropic human immunodeficiency virus type 1 isolate 89.6. J Virol. 2001;75(19):9177-86.

91. Berenbaum MC. What is synergy? Pharmacol Rev. 1989;41(2):93-141.

92. Greco WR, Bravo G, Parsons JC. The search for synergy: a critical review from a response surface perspective. Pharmacol Rev. 1995;47(2):331-85.

93. Geary N. Understanding synergy. Am J Physiol Endocrinol Metab. 2013;304(3):E237-53.

94. Grabovsky Y, Tallarida RJ. Isobolographic analysis for combinations of a full and partial agonist: curved isoboles. J Pharmacol Exp Ther. 2004;310(3):981-6.

95. Chou TC. Theoretical basis, experimental design, and computerized simulation of synergism and antagonism in drug combination studies. Pharmacol Rev. 2006;58(3):621-81.

96. Tallarida RJ, Raffa RB. Testing for synergism over a range of fixed ratio drug combinations: replacing the isobologram. Life Sci. 1996;58(2):PL 23-8.

97. West AP, Jr., Scharf L, Horwitz J, Klein F, Nussenzweig MC, Bjorkman PJ. Computational analysis of anti-HIV-1 antibody neutralization panel data to identify potential functional epitope residues. Proc Natl Acad Sci U S A. 2013;110(26):10598-603.

98. Ward JH. Hierarchical grouping to optimize an objective function. Journal of the American Statistical Association. 1963;58(301):236-44.

**Supplementary Figures:**

**Fig. A.**

**Fig. A.** Hierarchical clustering of IC_50_ (A, B) and IC_80_ (C, D) titers for panel bnAbs. Hierarchical clustering was performed using either Log10 IC_50_ or IC_80_ titers (A, C) or binary variables for sensitive/resistant viruses (B, D). “< Threshold” indicates positive responses and “>= threshold” indicates negative responses. Clustering trees show bootstrap values calculated using 100 bootstrap realizations. Calculations were performed using the Heatmap tool on Los Alamos HIV Database with Ward clustering option [98].

**Fig. B.**

**Fig. B: Prediction of IC_50_ and IC_80_ titers using the Additive, BH, and no-overlap BH models for bnAb combinations targeting similar epitopes.** Two bnAb combinations, where both bnAbs targeted the same epitope (CAP256-VRC26.25 + PGDM1400 and PGT128 + 10-1074V) were experimentally tested against 20 viruses. Predictions from the Additive, BH, and no-overlap BH models are shown as compared to the experimentally measured IC_50_ and IC_80_ values. Only viruses with IC_50_ and IC_80_ values within the experimental concentration range are shown. The no-overlap BH model was found to be significantly better than the BH model for each comparison (data not shown). Statistical comparison was performed between Additive and no-overlap BH model predictions using Wilcoxon paired rank test on absolute differences between predicted and observed Log_10_ IC_50_ and IC_80_ scores.

**Fig. C.**

**Fig. C: Comparison of Additive and BH models for predicting bnAb combination neutralization scores for Kong et al. panel.** Additive and BH models were used to model bnAb combination IC_80_ scores for the Kong et al. datasets [60]. In (A), Bliss model predictions are plotted against observed IC_80_ values for 125 viruses, with different bnAb combinations (n=22, two datasets of 11 combinations) shown by different colors and/or symbols. In (B), median Log10 deviations are shown between predicted and observed IC_80_ scores for different types of bnAb combinations, using Bliss model (blue) and Additive model (green). Statistical significance was calculated for each type of bnAb combination using Wilcoxon paired rank test on paired absolute Log10 differences between observed and predicted IC_80_ titers for all tested viruses, and indicated p-values were below the False Discovery Rate threshold q < 0.1.

**Fig. D.**

**Fig. D: Patterns in Additive and BH model predictions for IC_80_ titers for Clade C panel.** This figure shows the additive and BH model predicted IC_80_ titers for the ten 2-4 bnAb combinations from this study. In each panel the viruses are ordered using the potency of observed IC_80_ titers for the given combination. The additive model predicted titers were higher than the observed for most combinations, while for BH model predicted were closer to the observed. The accuracy of predictions between additive and BH model was compared using a Wilcoxon paired rank test on absolute Log_10_ deviations between observed and predicted IC_80_ titers, and only those p-values that corresponded to a false discovery rate of q < 0.1 are shown.

**Fig. E.**

**
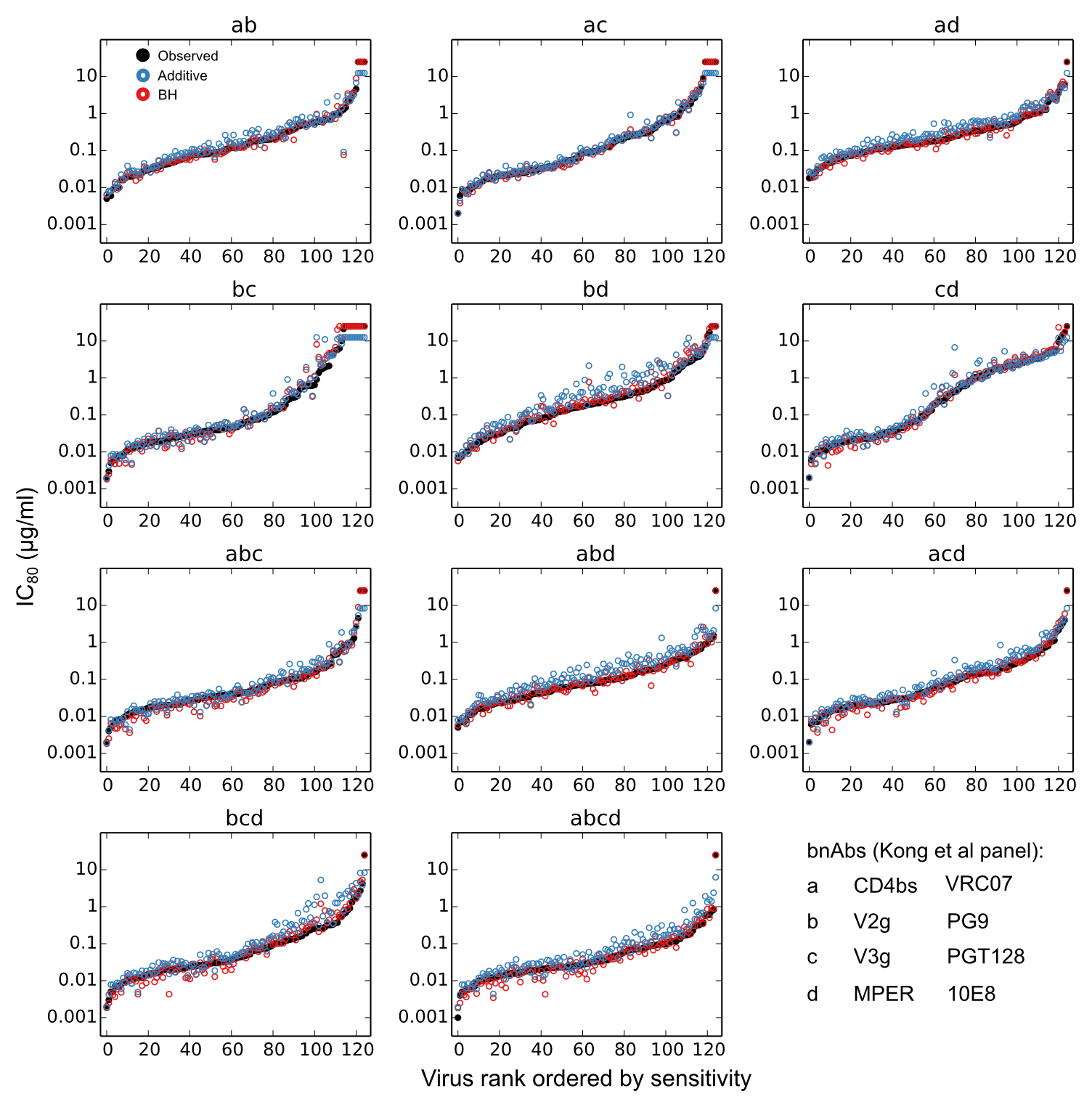
**

**Fig. E: Patterns in Additive and BH model predictions for IC_80_ titers for Kong et al panel.** This figure shows the additive and BH model predicted IC_80_ titers for the 11 2-4 bnAb combinations in Kong et al dataset 1. In each panel the viruses are ordered using the potency of observed IC_80_ titers for the given combination. The additive model predicted titers were higher than the observed for most combinations, while for BH model predicted were closer to the observed.

**Fig. F.**

**Fig. F: Modeling deviations between observed and BH model predicted IC_80_ scores.** Deviations, as defined by Log_10_ (Predicted IC_80_) – Log_10_ (Observed IC_80_), are shown for the Kong et al. datasets 1 (A) and 2 (B) (1), as a function of median corrected BH-predicted IC_80_ values on the x-axes. Only viruses that were neutralized by at least two bnAbs in the combination are considered. Modeling of these deviations is discussed in Supplementary Information, *“Modeling Systematic Deviations from Bliss-Hill Model predictions”*. The Kong et al. data comprises of two analogous datasets, and the model learnt for a given combination from one dataset (e.g. VRC07 + PGT128) was tested for its fit for the analogous combination in the other dataset (e.g. 3BNC117 + 10-1074). In each panel, BH model prediction (horizontal blue line at y=0), best-fit models for the same dataset (yellow lines) and best-fit models from the other dataset (red lines) are also shown. Indicated in each panel are also whether BH model provided the best-fit for the patterns of deviation (blue text), or whether modeling from other dataset significantly improved predictions over BH model (red text). In the latter case, statistical comparisons were performed using Wilcoxon Paired rank test on the absolute differences in Log_10_ IC_80_ between the observed and predicted values, predicted using BH model with and without deviation modeling from other dataset. Only p-values that had q-values < 0.1 are shown.

**Fig. G.**

**Fig. G: Using similar patterns in deviations of combination IC_80_ values from BH predictions to improve predictions.** Deviation models for the V2g+MPER and CD4bs+V2g+V3g combinations from Kong et al. dataset (1) are shown (A and B, D and E). The yellow line indicates the best fit for each combination in each of the Kong et al. data. Since Kong et al. data comprises of two analogous datasets, we also investigated how well the deviation model learnt for a given combination (e.g. VRC07 + PGT128) from one dataset fit the analogous combination (e.g. 3BNC117 + 10-1074) in the other dataset (depicted by red lines). The blue line is a flat line at y=0, indicating the BH model prediction. These deviation models were used to predict the combination IC_80_ scores for similar combinations in Clade C panel (C and F) (Supplementary Information, *“Modeling systematic deviations from the Bliss-Hill model predictions”*). In C and F, the observed IC_80_ values for the Clade C panel combinations are shown as grey points, and the BH model predictions are shown as open blue points. The predictions using BH and deviation modeling from the Kong et al. datasets are shown as open red points. For each of these combinations, deviation modeling improved predictions (significantly for V2g + MPER combination, and non-significantly for CD4bs+V2g+V3). Statistical significance was calculated using Wilcoxon paired rank test on absolute differences in observed and predicted Log_10_IC_80_ values, using BH model and using BH and deviation modeling. CAP256.25 = CAP256-VRC26.25.

**Fig. H.**

**Fig. H: Potency-breadth curves with and without deviation modeling for the best-in-category bnAb combinations.** Deviation modeling learnt from Kong et al. datasets was used together with BH model (“BH + DM”) to predict IC_80_ scores for the combinations of the type V2g + MPER, CD4bs + V2g + V3g and V2g + V3g + MPER (Supplementary Information, “Modeling systematic deviations from Bliss-Hill model predictions”). These scores were used to calculate the full potency-breadth (PB) curves for the best-in-category 2 (A), 3 (B) and 4 (C) bnAb combinations with (dark dotted lines) and without (solid, light lines) deviation modeling. For (C), deviation modeling for CD4bs + V2g + V3g was used for the 4 bnAb combinations that in total targeted these three epitopes.

**Fig. I.**

**Fig. I: Potency-breadth curves using additive model predicted IC_80_ for the best-in-category bnAb combinations.** Potency breadth curves are shown for best-in-category 2 (A), 3 (B) and 4 (C) bnAb combinations using IC_80_ scores predicted by additive model (dark) and BH model (faint). (D) shows the ordering of best-in-category combinations using additive model predicted geometric mean IC_80_ scores, and if combinations had less than 0.001μg/ml difference in geometric means, they are considered equivalent (denoted by ‘~’).

**Fig. J.**

**Fig. J: Extent of neutralization by multiple active bnAbs from best-in-category combinations at high concentrations.**  Similar to Fig. 5 except with single antibody activity IC_80_ thresholds of < 50 µg/ml (top row) and 100 µg/ml (bottom row). Since the maximum concentrations assayed were lower than these high concentrations, the single bnAb neutralization curves were extrapolated using modeling (see Supplementary Information, “Curve fitting for experimental single bnAb neutralization curves”). It should be noted that while these concentrations approximate the 28-day trough plasma concentrations of passively administered 3BNC117 in human trials and lead to transient drops in plasma viremia [57], it is not clear yet whether these high concentrations can be maintained in relevant tissues.

**Fig. SK.**

**Fig. K: Modeling maximum percent inhibition (MPI) of bnAb combinations using single bnAb MPI values.** This figure shows the observed and predicted MPI values for the 2, 3 and 4 bnAb combinations from Kong et al. dataset 1. The experimental MPI values were derived from assays with maximum starting concentrations of 25μg/ml, and the predicted MPI values were derived from using Bliss model on experimental single bnAb MPI values at 25 μg/ml.

**Fig. L.**


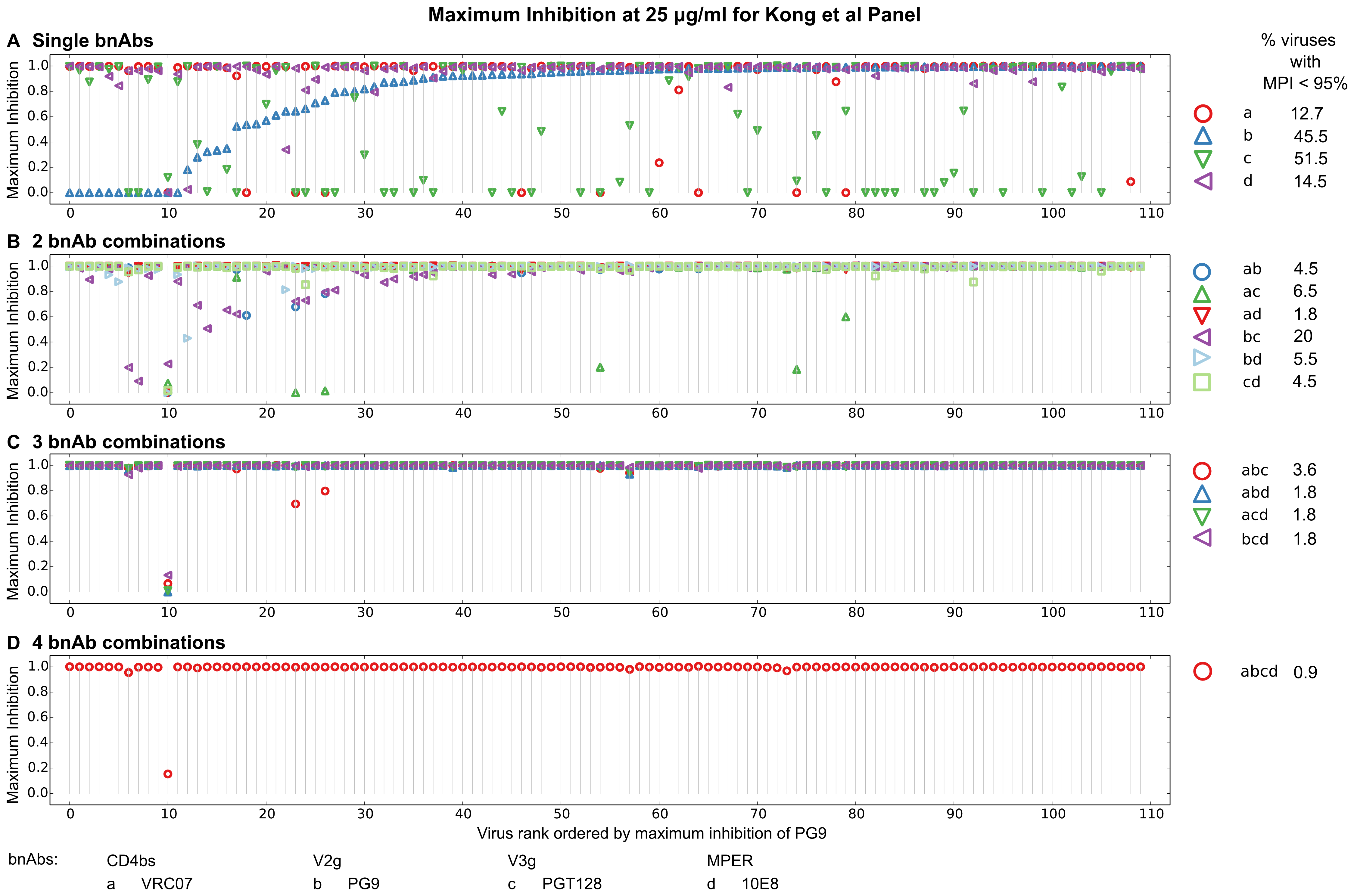


**Fig. L: MPI for bnAbs and bnAb combinations at 25 μg/ml for Kong et al. dataset 1.** Same as Fig. 6, except using observed experimental MPI at 25 μg/ml for single bnAbs and bnAb combinations from the Kong et al. dataset 1 [60].

**Fig. M.**

**Fig. M: Single bnAb neutralization curves for experimental assays with higher starting concentrations.** This figure shows the experimental neutralization curves for the best-in-class bnAbs against viruses that showed incomplete neutralization at starting concentrations of 20 or 25 μg/ml (faint curves). For these cases, additional assays were performed with higher starting bnAb concentrations (100-200 μg/ml) and these experimental curves are shown in dark colors. Even at higher starting concentrations, most of the bnAb-virus combinations tested showed incomplete neutralization.

**Fig. N.**

**Fig. N: Prediction of MPI at higher bnAb concentrations. (**A) Best-fit Hill models were determined using neutralization curves up to 25 µg/ml for the high starting concentration experimental assays in Fig. K. These best-fit models were used to predict the neutralization at 100 or 200 μg/ml and these values were compared to the observed neutralization values. The statistical significance of the observed and predicted values was analyzed using Kendall Tau test and the p-value is indicated. The error bars indicate standard deviation in predictions using error modeling (Supplementary Information, *“Error modeling for curve-fitting of single bnAb neutralization curves”*) and the chi-squared value was calculated using these values. (B) The distribution of predicted neutralization values at 100 μg/ml is shown for the best-in-class bnAbs for all of the clade C viruses using the modeling approach as in (A), with the fraction of viruses with less than 95% neutralization indicated below each bnAb.

**Fig. O.**

**
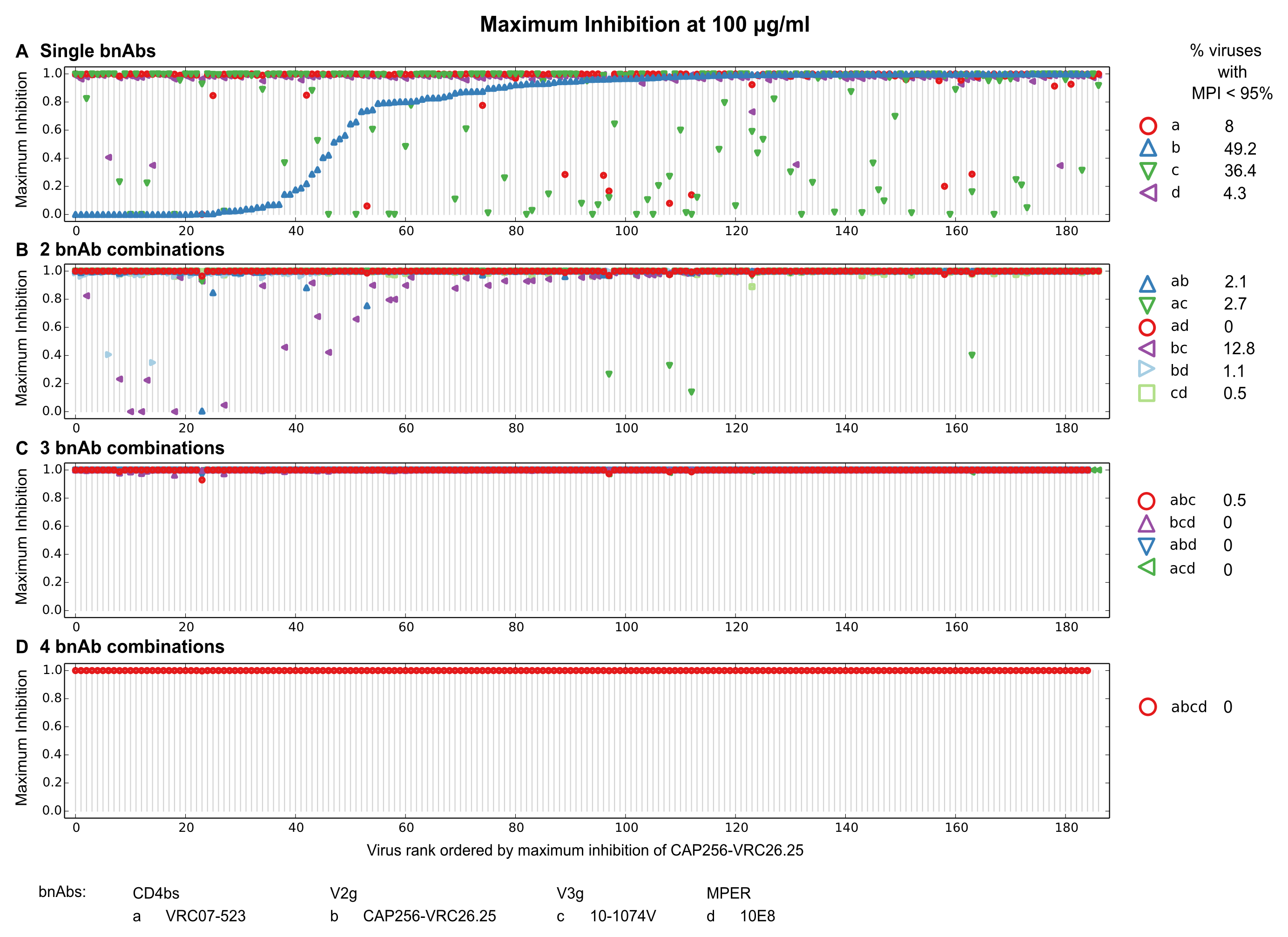
**

**Fig. O: Predicted MPI for single bnAbs and bnAb combinations at 100 μg/ml.** Same as Fig. 6, except using predicted MPI values at 100 μg/ml.

**Fig. P.**

**Fig. P: Instantaneous Inhibitory Potential (IIP) for clade C panel bnAbs.** IIP is defined as the Log_10_ fold reduction in the number of infectious events. The neutralization functions for each virus and bnAb were assumed to be Hill models, with IC_50_ and IC_80_ values used to determine the parameters (Supplementary Information, “*Curve fitting* *for experimental single bNAb neutralization curves*”). These functions were used to calculate IIP values at 1, 10 and 100 μg/ml. The horizontal dashed lines indicate an IIP of 5 Log_10_, a threshold that correlates with clinical success of antiretroviral drug combinations [75].

**Table S1. Breadth and potency of individual bnAbs versus clade C panel**

**Table A. Breadth and potency of individual bnAbs versus clade C panel**

**Table B. Breadth of neutralization by multiple active bnAbs from best-in-category combinations.**

|  | **Best % Coverage (IC_80_)** | | | **Best % Coverage (IC_50_)** | | |
| --- | --- | --- | --- | --- | --- | --- |
| **# of bnAbs in combination** | **2 or more**  **bnAbs active** | **3 or more**  **bnAbs active** | **4 or more**  **bnAbs active** | **2 or more**  **bnAbs active** | **3 or more**  **bnAbs active** | **4 or more**  **bnAbs active** |
|  | **IC_80_ < 1 μg/ml** | | | **IC_50_ < 1μg/ml** | | |
| 2 | 44 |  |  | 64.5 |  |  |
| 3 | 67.5 | 19.5 |  | 87 | 44.5 |  |
| 4 | 73.5 | 26.5 | 2.5 | 95 | 68.5 | 27.5 |
|  |  |  |  |  |  |  |
|  | **IC_80_ < 5 μg/ml** | | | **IC_50_ < 5 μg/ml** | | |
| 2 | 57 |  |  | 91.5 |  |  |
| 3 | 80.5 | 36.5 |  | 97 | 65.5 |  |
| 4 | 89.5 | 62.5 | 17.5 | 99.5 | 87 | 40 |
|  |  |  |  |  |  |  |
|  | **IC_80_ < 10 μg/ml** | | | **IC_50_ < 10 μg/ml** | | |
| 2 | 80 |  |  | 92.5 |  |  |
| 3 | 91 | 49.5 |  | 97.5 | 66.5 |  |
| 4 | 95.5 | 77 | 26 | 100 | 89 | 40.5 |
|  |  |  |  |  |  |  |
|  | **IC_80_ < 50 μg/ml** | | | **IC_50_ < 50 μg/ml** | | |
| 2 | 93 |  |  | 93 |  |  |
| 3 | 97.5 | 68 |  | 98 | 68.5 |  |
| 4 | 100 | 90.5 | 41.5 | 100 | 91 | 44 |
